# Genome Sequences of Akhmeta Virus, an Early Divergent Old World Orthopoxvirus

**DOI:** 10.3390/v10050252

**Published:** 2018-05-12

**Authors:** Jinxin Gao, Crystal Gigante, Ekaterine Khmaladze, Pengbo Liu, Shiyuyun Tang, Kimberly Wilkins, Kun Zhao, Whitni Davidson, Yoshinori Nakazawa, Giorgi Maghlakelidze, Marika Geleishvili, Maka Kokhreidze, Darin S. Carroll, Ginny Emerson, Yu Li

**Affiliations:** 1 Poxvirus and Rabies Branch, Division of High-Consequence Pathogens and Pathology, National Center for Emerging and Zoonotic Infectious Diseases, Centers of Disease Control and Prevention, 1600 Clifton Road NE, Atlanta, GA 30333, USA; jgao2@cdc.gov (J.G.); lzu1@cdc.gov (C.G.); pliu0628@gmail.com (P.L.); Tangshi06@gmail.com (S.T.); ibx4@cdc.gov (K.W.); vzt5@cdc.gov (K.Z.); wfd6@cdc.gov (W.D.); inp7@cdc.gov (Y.N.); DCarroll@cdc.gov (D.S.C.); GEmerson@cdc.gov (G.E.); 2 Laboratory of Molecular Epidemiology, National Center for Disease Control and Public Health of Georgia, 9 M. Asatiani Street, Tbilisi 0177, Georgia; e.khmaladze@ncdc.ge; 3 Division of Global Health Protection (DGHP), Center for Global Health, Centers for Disease Control and Prevention, 1600 Clifton Road NE, Atlanta, GA 30333, USA; kuo9@cdc.gov (G.M.); ivu9@cdc.gov (M.G.); 4 Laboratory of the Ministry of Agriculture of Georgia (LMA), Animal Disease Diagnostic Department, 49 Vaso Godziashvilis Street, Tbilisi 0159, Georgia; maka.kokhreidze@lma.gov.ge

**Keywords:** Akhmeta virus, orthopoxvirus, cowpox virus, recombination

## Abstract

Annotated whole genome sequences of three isolates of the Akhmeta virus (AKMV), a novel species of orthopoxvirus (OPXV), isolated from the Akhmeta and Vani regions of the country Georgia, are presented and discussed. The AKMV genome is similar in genomic content and structure to that of the cowpox virus (CPXV), but a lower sequence identity was found between AKMV and Old World OPXVs than between other known species of Old World OPXVs. Phylogenetic analysis showed that AKMV diverged prior to other Old World OPXV. AKMV isolates formed a monophyletic clade in the OPXV phylogeny, yet the sequence variability between AKMV isolates was higher than between the monkeypox virus strains in the Congo basin and West Africa. An AKMV isolate from Vani contained approximately six kb sequence in the left terminal region that shared a higher similarity with CPXV than with other AKMV isolates, whereas the rest of the genome was most similar to AKMV, suggesting recombination between AKMV and CPXV in a region containing several host range and virulence genes.

## 1. Introduction

Poxviruses are large double-stranded DNA viruses with genome sizes ranging from 140 to 280 kb that replicate exclusively in the cytoplasm of infected cells. The Poxviridae family is divided into two subfamilies, *Entomopoxvirinae* and *Chordopoxvirinae,* which cause infections in insects and vertebrates, respectively. *Chordopoxvirinae* is further divided into eight genera, among which *Orthopoxvirus* (OPXV) as well as *Parapoxvirus, Yatapoxvirus*, and *Molluscipoxvirus* contain species that cause human diseases. OPXV consists of 10 recognized species: *Camelpox virus* (CMLV), *Cowpox virus* (CPXV), *Ectromelia virus* (ECTV), *Monkeypox virus* (MPXV), *Taterapox virus* (TATV), *Vaccinia virus* (VACV), *Variola virus* (VARV), *Racconnpox virus* (RNCV), *Skunkpox virus* (SKPV), and *Volepox virus* (VPXV). RCNV, SKPV, and VPX are known as the New World OPXVs and the rest as Old World OPXVs. Immunity originating from one Old World OPXV species provides cross-protection against infections caused by other species [1]. *Variola virus*, the causative agent of smallpox, is the most devastating infectious agent in human history, killing up to 500 million people during the 20th century alone [2]. In 1980, smallpox was officially declared eradicated through a global vaccination program led by the World Health Organization; consequently, the vaccination program ceased.

After the eradication of smallpox, infections by non-VARV OPXVs have emerged worldwide. Recent reports of OPXV infections include cowpox-like cases in Europe [3], monkeypox in Central Africa [4], and in the U.S. [5], vaccinia infections in Brazil and Colombia [6,7], and camelpox and buffalopox in the Middle East, India, and Africa [8,9,10,11,12]. These infections represent the re-emergence of OPXVs, including cowpox-like viruses [3,13], monkeypox virus [14,15], vaccinia-like viruses [8,16,17] and camelpox virus [18,19,20]. Factors underlying virus re-emergence include the waning of smallpox vaccine-derived immunity, lack of public health resources (in some affected areas), political conflicts, and increased contact with small rodents or other mammalian hosts [21,22,23]. OPXV infections are zoonotic and arise from contact with rodents [24]; however, which species of rodents serve as reservoirs, how OPXVs circulate in rodents and other terrestrial mammals, and how they are transmitted to humans are poorly understood. These recently reported cases seem to represent only the “tip of the iceberg” [25,26].

In 2013, suspected cowpox infections were investigated in two men exposed to ill cows in the town of Akhmeta, Georgia (country), and the etiological agent was determined to be a novel OPXV species [25]. Subsequent serological investigation of humans and animals on the farm where the affected individuals worked uncovered the presence of anti-OPXV IgG titers in surrounding individuals, cows, and wild animals, indicating a naturally circulating OPXV in the area. In addition, a project for retrospective diagnosis of archived negative anthrax specimens, using a broadly reactive OPXV qPCR assay [27], uncovered a third virus isolate that caused a human infection in an area near the town of Vani, about 250 km from Akhmeta. DNA sequence analysis concluded that the three viruses belonged to the same species and were named Akhmeta virus (AKMV) [25].

An initial phylogenetic analysis using nine conserved genes indicated that this novel virus forms the deepest branch among all known Old World OPXVs [25]; a full genome sequence analysis will add new insight into the pathogenesis and evolution of AKMV and the OPXV genus. In addition, the AKMV isolates were detected by a monkeypox generic quantitative polymerase chain reaction (qPCR) assay, which had been previously validated for monkeypox specificity against all other available OPXV [28]. Whole genome sequences of these AKMV isolates will be useful in developing new monkeypox-specific qPCR assays and for validating other published OPXV generic and species-specific diagnostic assays.

## 2. Methods and Materials

### 2.1. Virus Genome Sequences

The OPXV genome sequences used in this study were retrieved from GenBank (http://www.ncbi.nlm.nih.gov/) and are listed in Supplementary Table S1. Sequences were imported into appropriate software for analysis as indicated below.

### 2.2. NGS Sequencing and De Novo Assembly

The two AKMV_AKHM13 isolates were passaged in BSC40 cells, and DNA was extracted from propagated virus, whereas DNA of the AKMV_VANI10 isolate was extracted directly from clinical samples. Genome sequencing was performed on the Illumina HiSeq 2000 platform at Otogenetics (Norcross, GA, USA). Reads were assembled using CLC Genomics Workbench 6.0. (QIAGEN, Aarhus, Denmark). Briefly, raw reads were imported into CLC, quality was assessed and low quality reads were removed including duplicate reads, reads with quality score <0.01, and reads with >2 ambiguous bases. Host reads were subtracted by mapping reads to an appropriate host reference in CLC, African Green Monkey genome, or human genome (HGC build 39). The unmapped reads were used in de novo assembly using default settings. The output contigs were screened for poxvirus matches using BLAST. Genomic positions and orientation of matched contigs were determined in reference to CPXV_BR. Draft genomes were constructed with four gaps that were closed using Sanger sequencing. Raw reads were mapped back to the gap-filled genome to inspect assembly accuracy.

### 2.3. Gap Closing

When mapped to the completed AKMV genome, gaps 1 to 4 were positioned at 103–04, 1025–1670, 8690–8710, and 97,738–97,826, respectively. Primer sets for closing these gaps were: G1F (5′-AGT GTC TAG AAA AAA ATG TGT GAC CAC-3′) and G1R (5′-GGA TAC TGC TCA CGT TTT TT-3′); G2F (5′- GGG GTG TTG GAT AAG CTT GA-3′) and G2R (5′-ACC TCT CGT TAC TTC TTC TT-3′); G3F (5′-CAG TTC TGT AAG AGA TGA GAA GCC TGT AGA-3′) and G3R (5′-AGC TCT AAT TGG ATG CGC TAT CTC TAA-3′); G4F (5′-ACG GAG AAC CAA TCA TTA TAA CAT CGT ATC TTC A-3′), and G4R (5′-CCT TAT TAT TGT TGG TAC GAG CCG TGT AAA C-3′). DNA sequencing was performed as previously described [29]. Briefly, 10–50 ng of total DNA was used in a 50 µL reaction using the Roche Expand Long Template PCR System (Roche, Cat 11681834001). The PCR program was run at 92 °C for 3 min, 30 cycles of 92 °C for 10 s, 50 °C for 30 s, and 68 °C for 90 s, and a final cycle at 68 °C for 4 min in an ABI GeneAmp PCR 9700 Thermocycler. PCR products were analyzed using agarose gel electrophoresis, and DNA from positive reactions were purified using the QIAquick PCR purification kit (Cat# 28106). Sequencing was performed from both directions using the BigDye^®^ Terminator v3.1 Cycle Sequencing Kit (ABI, Cat 4337035) in a 10 µL reaction volume containing 5–20 ng purified DNA template with a program at 95 °C for 1 min, then 25 cycles at 95 °C for 10 s, 53 °C for 10 s, and 60 °C for 4 min. Sequencing reactions were applied to an ABI 3130XL Genetic Analyzer. Called sequences were analyzed using Seqman in the Lasergene Package (DNASTAR, Madison, WI, USA). Consensus sequences were used to close gaps in the draft genomes.

### 2.4. Phylogenetic Analysis

The highly conserved central regions ranging from AKMV52 to AKMV155 (equivalent to F9L to A24R in VACV_CP) of 22 representative OPXVs were aligned using MAFFT (Multiple Alignment using Fast Fourier Transform) v7.313 [30]. The alignment was visually inspected for errors, which were manually corrected. Gap columns were removed and pairwise identity between sequences was recorded. The BEAST package (v2.4.7) was used for phylogenetic analysis [31]. Trees were constructed using the general time reversible (GTR) substitution model with 4 gamma rate categories and 10 M MCMC chain length.

### 2.5. Genome Annotation

Genes were annotated in Geneious software 7.0 (Biomatters Ltd, Auckland, New Zealand) [32] using a combination of annotation transfer and ORF finding. Gene annotations from two reference genomes, CPXV_BR and CPXV_GRI-90, were transferred to the AKMV isolate genomes when similarity was >70%. ORF-finding was performed using ORF > 120 nucleotides and ATG, TTG, or CTG as a start codon. When the predicted ORFs and transferred genes were superimposed, the ORFs were considered genes. Predicted ORFs that did not correspond to transferred genes were investigated using BLAST and were only considered genes when homologs existed in GenBank. Functions of the annotated genes were transferred from reference CPXV_BR; unannotated genes were investigated using BLAST search at the UniProt website (http://www.uniprot.org/) with an E-value setting of 0.0001. Protein identity analysis between AKMV_AKHM13-88 and reference proteins of CPXV_BR, CPXV_GRI-90, and ECTV_MOS was performed using local BLAST in BioEdit (http://www.mbio.ncsu.edu/BioEdit/bioedit.html). AKMV proteins were used as queries to BLAST against the proteome database of each reference virus, and BLAST output identities were reported.

### 2.6. Tandem Repeat Analysis in the Inverse Terminal Repetitions (ITR) of AKMV Genome

Tandem repeats were determined using the program Tandem Repeat Finder [33]. The scoring parameters were set to 2, −7, −7 (match, mismatch, deletion) and reporting score was set to 40. Percent matches were >90%. Fractions indicate the presence of an incomplete copy.

### 2.7. Genome and Protein Comparison

Alignment of genome sequences excluding the ITR was conducted using MAFFT. For easy comparison, the alignment was divided into three regions: the left terminal region, the highly conserved central region, and the right terminal region. Specifically, the left and right terminal regions ranged from *AKMV007* to *AKMV051* and *AKMV156* to *AKMV213*, respectively, as described previously [34]. The remainder, from *AKMV52* to *AKMV155*, was the conserved central region. In the conserved central regions, AKMV was compared to two ECTVs (ECTV_MOS and ECTV_NAV) and eight CPXVs (HumGra07/1, RatKre08/2, JagKre08/2, *HumMag07*/1, GER91, AUS99, CPXV_GRI-90, and CPXV_BR). At terminal regions, AKMV was compared to the two ECTVs and four CPXVs (CPXV_BR, CPXV_GRI-90, CPXV_GER91, and CPXV_AUS99) due to incomplete sequences. Only differences common to all compared CPXVs were counted. The same criteria were applied to the comparison with ECTV. Pairwise sequence identity with or without gaps was reported using Geneious 7.0. The cut-off value for gene truncation was equal to or greater than 10% of the protein length or >40 aa using AKMV genes as reference. Gene function comparison was limited to proteins that possessed homologs of known function.

### 2.8. Determination and Comparison of Host Range Factors

Sequences of the reported host range factors of CPXV_BR and GRI-90 [35] were retrieved from GenBank and were used as queries to search the proteome of AKMV_Akm13_88. Pairwise alignment of CPXV_BR factors and the corresponding AKMV homologs was conducted using MAFFT in the Geneious program.

### 2.9. Investigation of a Recombination Event in the AKMV_VANI10 Genome

A recombination event in AKMV_VANI10 was investigated using the Simplot program v3.5 [36]. Selected sequences were AKMV_VANI10, AKMV_AKHM13-88, and five CPXVs, each representing a CPXV monophyletic groups defined by Mauldin et al. [37]. AUS99 (HQ407377), GRI-90 (X94355), HumLit08/1 (KC813493), HumGra07/1 (KC813510), and CPXV_BR (NC_003663) were representative of the CPXV monophyletic groups A, B, C, D, and E, respectively. The regions from C1L to C9L (according to GRI-90) of the selected sequences were aligned using MAFFT. Gaps and ambiguous nucleotides were stripped and the resultant alignment was used as input for the Simplot program. AKMV_VANI10 sequence was set as the query and Simplot analysis was run using the F84 distance Model, Ts/Tv ration 2.0, with a sliding window size of 200 bp with a 20-bp step size and 250 bootstraps.

### 2.10. Analysis of dN/dS

The dN/dS ratio was examined for a subset of genes located within or adjacent to the region of suspected recombination in the AKMV_VANI10 genome. Coding regions of CPXV_GRI-90 C4L–C9L and C13L and C14L orthologs were extracted. CPXV_GRI-90 C2L and C3L were not used due to deletions in the AKMV_VANI10 ortholog. Each set of orthologs was aligned based on translation by MAFFT [30,38] using the FFT-NS-i x1000 algorithm and BLOSUM62 scoring matrix in Geneious. The tree file used for all dN/dS analyses was generated using an alignment of the DNA polymerase coding region by maximum likelihood based on the GTR+G model in Mega7 [39].

To identify genes under positive selection, dN/dS was examined using site models (model = 0, NSsites = 0, 1, 2, 7, 8) in the CODEML package in PAML [40]. Log likelihood tests were performed to determine if a model that included codons under positive selection fit the alignment for a given gene better than a model that did not allow positive selection (comparison of model M1a to M2a). In all cases, Model M1a exhibited significant improvement over model M0 by log likelihood ratio test. Sites where dN/dS > 1 were identified based on Bayes Empirical Bayes (BEB) analysis [41] using Pr(ω > 1) > 0.95 as a cutoff.

## 3. Results

### 3.1. AKMV Genome Assembly and ITR

The raw Illumina reads of the three AKMV isolates, AKMV_AKHM13-85, AKMV_AKHM13-88, and AKMV_VANI10, were assembled individually using de novo assembly in CLC Bio Genomics Workbench (v5). Five contigs were found for each isolate, although the average coverage of contigs varied between isolates (~1000 for the two AKMV–AKHM13 and ~100 for AKMV_VANI10). Gaps in the two AKMV_AKHM13 isolates were closed using Sanger sequencing, and filled sequences revealed that gaps were caused mainly by tandem repeats. The ITR region contained two gaps: the more terminal gap (Gap1) contained an 86-bp tandem repeat of 6.3 copies, and the inner gap (Gap2) had 11.5 copies of a 60-bp repeat. The gap in the central region (Gap4) contained a tandem repeat of 7 bp 18.7 times. The two junctions of the ITRs (Gap3) to the terminal regions also produced gaps without tandem repeats. The completed AKMV_VANI10 genome still contains two gaps that contain repetitive sequences that were not able to be filled due to the limited quantity of the DNA.

The completed genomes of AKMV_AKHM13-88 and AKMV_AKHM13-85 were 221,911 bp and 221, 902 bp, respectively. The starting A of the conserved concatemer resolution sequence, 5′-ATTTA-N7-9-A6-7-3′ [42,43,44], was set as position 1 for both genomes. The G + C content of the completed AKMV genomes contained 66.5% A + T in nucleotide composition, similar to those of other OPXVs [34,45]. The ITRs of the two AKMV_AKHM13 isolates were identical with a length of 8725 bp. There are four differences between the complete AKMV_AKHM13-85 and AKMV_AKHM13-88 genomes, consisting of three SNPs and one six-nucleotide indel. These slight differences are consistent with the fact that the two cases are closely linked. For simplicity, AKMV_AKHM13-88 was used in all further analyses. The assembled AKMV_VANI10 genome was 217,740 bp with an incomplete ITR of 7047 bp. The start of the AKMV_VANI10 genome corresponds to position 1651 of AKMV_AKHM13-88. The AKMV_VANI10 genome contains two gaps: one located at position 19,280 with a tandem repeat of AC and the second located at position 131,462, containing a tandem repeat of the six nucleotides TTACTA. Each gap is filled with either NN or NNNNN due to uncertainty in the copy number. There are 2146 SNPs and 156 indels between the assembled AKMV_VANI10 and AKMV_AKHM13-88 genome sequences.

Gene annotation of the two AKMV_AKHM13 genomes revealed 220 potential genes that encode proteins ranging from 43 to 1926 aa (Supplementary Tables S2 and S3). Of these 220 genes, 217 shared substantial sequence identity to genes in CPXV_BR. All genes were found to have homologs in ECTV or CPXV, so AKMV does not possess any new protein families outside of known OPXVs.

**Figure 1:**
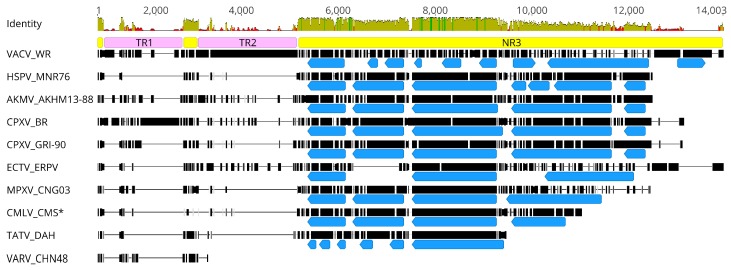
The ITR alignment of selected orthopoxvirus (OPXV) sequences. Alignment position is indicated at the top. Sequence conservation is shown with an identity graph (100% green, at least 30% and under 100% greeny-brown, under 30% red). Yellow boxes indicate non-repetitive (NR) unique sequences NR1, NR2, and NR3 from left to right, respectively. Faint purple boxes indicate tandem repeat (TR) TR1 and TR2. Blue boxes show the location of annotated genes with arrows pointing in the direction of transcription within the NR3. Black bars represent partially aligned genome sequences, horizontal lines represent alignment gaps; * missing the NR2. Detailed information about each ITR region is listed in Supplementary Table S4.

The size and structure of the AKMV_AKHM13 ITRs are comparable to that of CPXV except the length and the sequence of tandem repeats (Supplementary Figure 1 and Table S4). Those long tandem repeat (TR) sequences are usually preceded by non-repetitive unique sequences (NR) [46,47,48]. The first NR region (NR1) of AKMV_AKHM13-88 is a 78-bp sequence located at the tip of the ITR and contains the concatemer resolution sequence. BLAST search of the NR1 against OPXV in Genbank revealed that it is well conserved among Old World OPXV species, with nucleotide identity above 89.4%. The next NR region (NR2) of AKMV_AKHM13-88 (positions 683 to 982 bp) shares an identity of 84% or higher among Old World OPXVs. Between the NR1 and NR2 is the first set tandem repeat (TR1) region containing 6.3 copies of an 86-bp repeat. In other OPXVs, the TR1 varies in both repeat number and repeat sequence. For example, horsepox virus has 1.3 tandem repeats, whereas some VACV and CPXV have a large number of tandem repeats; CPXV_BR also contain a secondary tandem repeat in addition to the major repeat (177 bp). The second tandem repeat region (TR2) after the NR2 also contains repetitive sequences that are highly variable in size among OPXV species. AKMV_AKHM13-88 contains 11.5 copies of a 60-bp sequence. VACV_WR contains two types of tandem repeats and is among the largest ITR of all Old World OPXV species (Supplementary Table S4). The sequence after TR2 contains mostly unique sequences (NR3) with a small number of discrete short repeats (2–7 bases). The NR3 of AKMV is about 7.0 kb and mostly resembles that of CPXV, both in sequence similarity and gene content (Figure 1). This region demonstrated considerable size variation between AKMV and other species of OPXV due to gene deletions or truncation (Supplementary Table S2). Sequence variation goes beyond the ITR and spreads into terminal regions until the highly conserved central region.

### 3.2. Phylogenetic Analysis of AKMV with Other OPXVs

**Figure 2:**
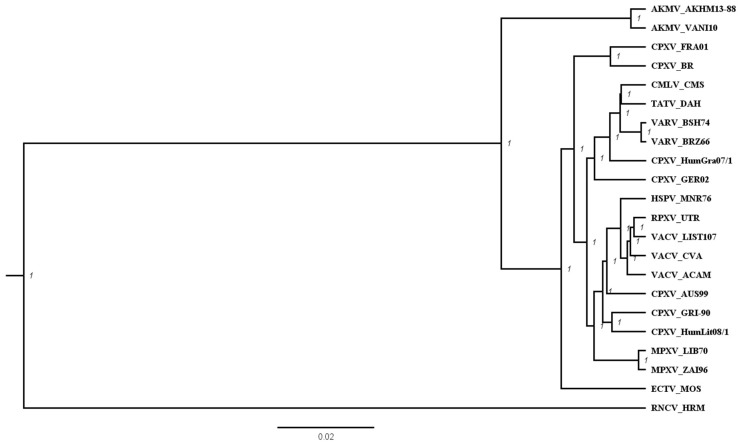
Phylogenetic analysis of OPXV species using the 100-kb central region of genome. Twenty-two selected OPXVs were aligned using MAFFT and gap columns were removed. A phylogenetic tree was constructed using BEAST (v2.4.7). Posterior probability is 1 at each node of the reconstructed tree, indicatingthe phylogeny is well supported. The tree was drawn in the scale of substitutions per site. The identity matrix table of these OPXV sequences is presented in Supplementary Table S5.

To study the phylogeny of AKMV at the genomic level, the ~100 kb highly conserved central region of 22 OPXVs were analyzed as previously reported [34]. The tree topologies constructed using BEAST (Figure 2) were essentially the same as the previously-reported nine-gene tree [25]. These trees indicated that AKMV diverged early among OPXVs in poxvirus evolution. AKMV isolates formed a separate clade lying between the New World and the Old World OPXVs. ECTV was positioned between AKMV and other Old World OPXV clades, consistent with the notion that ECTV used to be most diverged among the reported Old World OPXVs [45]. A sequence identity matrix of the 22 selected OPXVs is presented in Supplementary Table S5. The average identity among the selected Old World OPXV species (excluding AKMV) and between Old World and New World OPXVs is 97.65% and 86.63%, respectively, whereas the average identity AKMV shared with the Old World OPXVs and the New World OPXVs were 95.42% and 86.70%, respectively.

### 3.3. Genome Characteristics of AKMV

The AKMV genomes were aligned to those of two ECTVs and eight CPXVs. For easy comparison, the alignment was divided into three regions: the left terminal region, the central region, and the right terminal region. The central regions of the 11 OPXV genomes demonstrated high conservation both in length (<275 bp) and in identity (>95% with gaps removed) (Supplementary Table S3), with the longest genome being AKMV (99,175 bp) and the shortest genome CPXV_GRI-90 (98,901 bp). AKMV genes were in a co-linear arrangement with those of CPXV and ECTV, and protein identity ranged from 80–100%, with an average above 96%. The observed high conservation is consistent with a previous report detailing diversity among OPXV species [34]; however, minor differences did exist. Compared with all CPXVs, AKMV lacked only one small CPXV gene (*CPXV119A* in CPXV_BR, encoding an 80 aa protein), while it possessed one small gene *AKMV059* that is missing only in some CPXV isolates. AKMV displayed more differences with ECTV in that it encoded seven more small putative genes that are missing in ECTVs. Overall, the central region of AKMV is comparable to those of CPXVs with minimal gene difference.

Unlike the highly conserved central region, both terminal regions of AKMV displayed increased variation compared with CPXVs and ECTVs. Because genes in the terminal regions are involved in virulence and host-range determination [48,49], characterization of gene variations in these regions might help elucidate novel features of AKMV pathogenesis. Three large deletions were evident in ECTV sequences compared to AKMV in the alignment, and these deletions corresponded to the loss of nine AKMV homologous genes (*AKMV013-017, AKMV021, AKMV023-024,* and *AKMV029*). In addition, ECTV lacked another nine genes due to premature stop codons caused by small indels (Supplementary Table S2). In contrast, AKMV only lacked a homolog of one gene, *CPXV010* (of CPXV-BR) that was present in all CPXVs. Other genes missing AKMV were also missing or truncated in some CPXV isolates, so the presence of these genes appeared to be strain-dependent. For example, *CPXV013* of CPXV_BR is absent from AKMV and is also truncated in strain CPXV_AUS99.

Similar to the left terminal region, the right terminal region of AKMV also demonstrated reduced sequence identity (91%) and increased size variation. Two deletions in the ECTVs corresponded to the loss of four AKMV gene homologs of *AKMV198*, *AKMV199*, *AKMV212,* and *AKMV213* (Supplementary Table S2). In addition, ECTV lacked another 14 AKMV homologs due to premature stop codons caused by nucleotide mutations. In contrast, only minor indels were noticeable between CPXVs and AKMV in the alignment, and gene differences were observed to be strain-dependent (Supplementary Table S2). For example, although the homologs of *AKMV157* are fragmented in strain CPXV_BR, they are intact in CPXV_GRI-90. An extra 5.7 kb insertion was found present in CPXV_GER91, but not in other CPXVs or AKMV. At the right ITR junction, AKMV lacks the *CrmD* that is also missing from GER91. Overall, AKMV shared the same number genes with CPXVs with some strain-dependent difference, as in the left terminus.

### 3.4. A Closer Look at Genes Differing between AKMV and CPXV_BR

Although the genomic content of AKMV is highly similar to that of CPXVs, AKMV did contain unique genomic features when compared with single CPXV isolates. For instance, 25 AKMV genes were markedly different from the well-characterized CPXV-BR at the genomic termini (Supplementary Table S6), among which 13 genes were only present in one virus, and the remaining 12 genes showed different truncation or fragmentation between the two viruses. In this section, only genes having homologs of known function are discussed.

AKMV lacked homologs of CPXV_BR *CPXV013* and *CPXV221*. The *CPXV013* homolog in CPXV_GRI-90 (*D11L*) was reported to be involved in host range and virulence in combination with other CPXV kelch-like proteins [50]. It encodes a 524 aa protein that contains a N-terminal POZ (poxvirus and zinc-finger) domain and three kelch domains. *CPXV221* encodes CrmD (320 aa), a decoy TNF-alpha receptor [51,52]. On the other hand, AKMV maintained two putative virulence genes that were absent from CPXV_BR, *AKMV007* and *AKMV213*. AKMV007, similar to CPXV_GRI-90 D7L, contains a kelch repeat and a POZ domain that are thought to modulate host ubiquitin-protein ligase activity. AKMV214 is similar to CPXV_GRI-90 K3R, which is a receptor for host TNF and other chemokines [51].

Six AKMV genes were truncated compared to their homologs in CPXV_BR, of which three are predicted to function in virulence. First, *AKMV156* encodes an ATI protein with an inner 55 aa deletion and 20 aa deletion at the C-terminal region compared to the CPXV ATI. In addition, its C-terminal 140 aa shared low identity (22%) with CPXV_BR homolog. CPXV ATI protein forms a matrix with other proteins that surround the mature virion to protect it from harsh environments. However, *ATI* is disrupted in many Old World OPXVs. Second, *AKMV182* is homologous to *CPXV186*, but its product lacks the N-terminal 24 aa that contains the nucleotide-biding P-loop domain. *CPXV186* encodes thymidylate kinase (TMPK) that is considered a target for anti-variola drug development [53,54,55]. Third, *AKMV211* has a 3′ terminal truncation that makes it half the size of its homolog, *CPXV218,* that encodes a chemokine-binding protein.

Two genes, *AKMV009* and *AKMV198,* were intact in *AKMV* but homologs were truncated in CPXV_BR. *AKMV009* encodes a 159 aa of type II membrane protein with a C-type lectin-like domain at its C-terminus. Its counterpart in CPXV_BR was reduced to one smaller gene *CPXV012* (encoding a 69 aa protein). CPXV012 was reported to bind transporter associated with antigen processing (TAP) on the endoplasmic reticulum (ER) membrane to block viral antigen transportation into ER lumen, thus preventing viral peptide from being displayed on cell surfaces of infected cells [56]. This function of CPXV012 was mapped to a unique internal deletion that does not exist in AKMV009. Outside of poxvirus proteins, AKMV009 shared the highest amino acid identity with an uncharacterized Prairie vole protein, XP_013209957 (44%), and a moderate identity (37%) with the rat cytomeglovirus C-type lectin-like (RCTL) protein, a decoy ligand to the natural killer inhibitory receptor (P1B) [57] (Supplementary Figure S1). It raises the possibility that AKMV009 may have the same function as RCTL. AKMV198 contained 23 more amino acids at its N-terminus than its CPXV_BR counterpart, *CPXV203*. It was reported that *CPXV012* and *CPXV203* work in the same pathway to block the viral antigen presentation to cell surfaces through different mechanisms [58,59]. Further examining how these closely related proteins work in related viruses would be interesting future work.

To shed light on the AKMV host range, the reported CPXV host range factors [35,60] were mapped to the AKMV_AKHM13-88 proteome using a similarity search. The results demonstrated that AKMVs shared a profile similar to CPXV_BR (Supplementary Table S7). Both viruses had 26 host range factors that covered all families of host range factors. Considering the average nucleotide sequence identity (90%) of terminal regions, 11 of the 26 AKMV factors demonstrated the same level identity at the protein level and shared a consistent length with corresponding CPXV factors. However, the remaining 15 factors demonstrated some differences, including complete absence, indel, low identity, and truncation. For example, AKMV lacked CrmD while containing CrmE, compared to CPXV_BR, which contains CrmD and not CrmE. Additionally, CrmC of AKMV had a short N-terminal truncation compared to its CPXV counterparts.

### 3.5. Investigation of a Recombination Event in AKMV_VANI10 Genome

**Figure 3:**
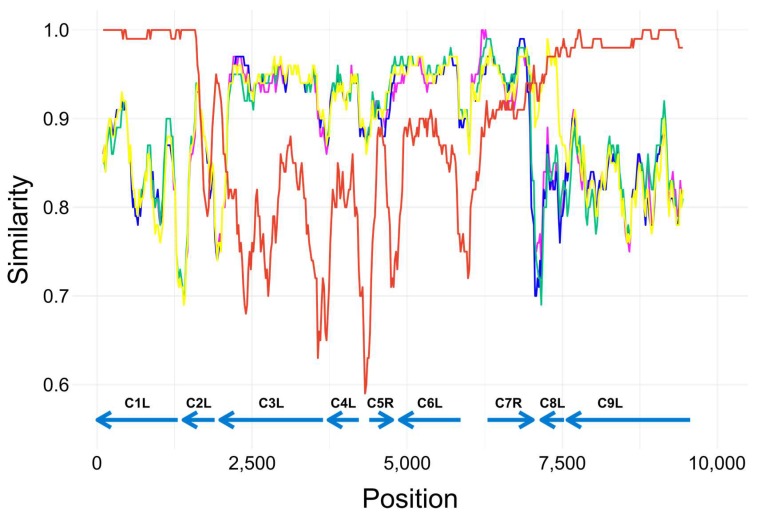
A recombination event in the AKMV_VANI10 sequence was investigated using Simplot. The identity of AKMV_VANI10 to each of the selected sequences was plotted on the Y-axis, and the X-axis indicates the position of alignment. Each colored line represents one virus. Orange, AKMV_AKHM13-88; pink, CPXV_AUS99; yellow, CPXV_GRI-90; blue, CPXV_HumLit08/1; cyan, CPXV_HumGra07/1; and green, CPXV_BR. Arrows indicate positions of coding regions.

Whereas the overall sequence similarity between AKMV_VANI10 and AKMV_AKHM13 was 99.4%, an approximately 6.5 kb sequence in the left terminal region between AKMV014 (C2L of CPXV_GRI-90) and AKMV020 (C8L of CPXV_GRI-90) displayed dramatically reduced sequence similarity of 86%. BLAST search using the six kb of AKMV_VANI10 as query showed this region was 94% identical to CPXV sequences. Simplot analysis showed that the identity cross-over points lie in the C2L and C7R–C8L genes, strongly supporting that this unique region of AKMV_VANI10 is the result of a recombination event between two parental strains of AKMV and CPXV (Figure 3). Simplot analysis also demonstrated that the coding regions maintained higher levels of similarity relative to the intergenic regions. More information about the sizes of coded proteins in each strain and the identity compared to AKMV_VANI10 are summarized in Supplementary Table S8.

We further examined this region to determine the possible effects that recombination could have on proteins encoded by CPXV_BR *CPXV018*/CPXV_GRI-90 *C2L*–*CPXV023/C7R*. The AKMV_VANI10 C2L and C3L homologs displayed the largest disruptions compared to their counterparts in CPXV or AKMV_AKHM13. *AKMV014* (*CPXV018/C2L*) contains a single nucleotide deletion following the start codon that introduces a frame shift, producing a stop codon after four amino acids. A predicted ORF starting with the atypical start codon TTG would encode a 167 amino acid protein with 80% identity to *AKMV014* (AKMV_AKHM13-88). Using only ATG as a start codon, ORF prediction revealed a shorter peptide (114 aa) with 96.5% amino acid identity to *AKMV014* (AKMV_AKHM13-88). These predicted proteins correspond to an 11 or a 64 amino acid N-terminal deletion.

*AKMV015* (homologous to *CPXV019/C3L*) exhibited a large internal deletion in the AKMV_VANI10 isolate that included several predicted ankyrin repeat domains, as well as a highly variable region (Supplementary Figure S2). The deletion corresponded to amino acids 89 to 316 in AKMV_AKHM13-88. The resulting protein was predicted to be 581 aa, compared to ~800 aa homologs in AKMV_AKHM13-88 and many CPXV isolates.

The four remaining genes within the putative recombination region (*CPXV020/C4L* to *CPXV023/C7R*) did not exhibit obvious, large deletions. These four genes and four surrounding genes (corresponding to CPXV_BR *CPXV024*, *CPXV025*, *CPXV029*, and *CPXV030*) were examined for sites undergoing selection using site models in CODEML (PAML package). Three of the eight genes examined showed evidence of having codons under positive selection in the OPXV lineage based on the results of log-likelihood ratio tests. These genes were *CPXV020/C4L*, *CPXV023/C7R*, and *CPXV029/C13L*; all three have known functions in host range and virulence in OPXVs (Supplementary Table S7). Both C4L and C7R are host range/virulence genes belonging to the C4L or P28-like family of host range factors. C13L is a member of the KilA-N/RING domain-containing C7L protein family.

Sites under positive selection (dN/dS > 1) were identified for each of the three genes, and the corresponding amino acids in AKMV_AKHM13-88 and AKMV_VANI10 are indicated in Supplementary Table S9. All three sites where dN/dS > 1 in *CPXV023/C7R* differed between the two AKMV isolates, whereas the sites under selection were identical in AKMV_AKHM13 and AKMV_VANI10 for CPXV020/C4L and CPXV029/C13L. Alignment of the protein sequences revealed a deletion of six amino acids from the AKMV_VANI10 *CPXV023/C7R* protein relative to the AKMV_AKHM13 homolog. This deletion was flanked by two of the sites under positive selection, between the KilA-N and RING domains (Supplementary Figure S3). Alignment of *CPXV023/C7R* orthologs revealed a shorter linker between the KilA-N and RING domains in Old World OPXVs, but additional amino acids in this region in AKMV_AKHM13 and New World OPXVs (Supplementary Figure S2).

## 4. Discussion

Traditionally, CPXV is considered an ancestral-like OPXV due to its large genome size and near complete repertoire of poxvirus genes. ECTV is a lab-derived strain that contains multiple genomic deletions compared with CPXV but it is one of most diverged OPXV. Phylogenetic analysis based on either partial or whole genome sequences of Old and New World OPXVs revealed that AKMV isolates form a deeper branch relative to CPXV, ECTV, and other known Old World OPXV, showing that AKMV is the most ancestral Old World OPXV currently known. Geographically, Akhmeta is near the edge of the European continent where CPXV is endemic, and increased sequence variation across AKMV genomes suggests that AKMV has circulated in the region for a long period. Georgia is a mountainous country; the greater Caucasus Mountain Range forms Georgia’s northern border and separates it from Russia, therefore separating AKMV from CPXV.

The genome sequences of AKMV_VANI10 and AKMV_AKHM13 also exhibit a large number of sequence changes when compared, even without considering the changes associated with the putative recombination with CPXV. There are approximately 1300 SNPs between AKMV2010 and AKMV2013. In comparison, there are about 700 SNPs between variola major and minor, and about 800 SNPs between the MPXV Congo basin and West Africa clades [61]. The large sequence divergence between AKMV_VANI10 and AKMV_AKHM13 may be due to isolation caused by the mountainous barriers within Georgia. The Likhi Range divides the country into eastern and western halves. AKMV_VANI10 was isolated from the western part of the country and AKHM13 is from the eastern-central part of the country, 250 km apart. A previous serological survey identified a large number of serum positive rodents [25]. Thus, the interplay between cows, human, and rodents requires further investigation. More virus isolates and further study of natural reservoir rodents may improve our understanding of the evolution of AKMV and OPXV in general.

Despite the high similarities with CPXVs, AKMV demonstrated unique genomic features compared with the prototype CPXV_BR. In the two terminal regions AKMV lacked 11 gene homologs and contained another 12 genes remarkably different from their CPXV_BR counterparts. Many of these genes have predicted functions as host chemokine receptors, host range factors, and virulence factors. In addition, another 32 AKMV proteins demonstrated less than 85% identity to CPXV homologs in the context of approximately 90% nucleotide identity. These proteins are likely the result of AKMV adaptation to its specific host environment.

The host range of a particular OPXV has been suggested to be correlated with the possessed number and categories of host range factors [35]. Known OPXV host range factors can be classified into 12 categories (Supplementary Table S7). Among Old World OPXV species, CPXVs have the widest host ranges and the largest number of host range factors, 27 for CPXV_GRI-90 and 26 for CPXV_BR, with host range factors occupying every category. In contrast, the narrow host range ECTV and VARV each typically contain only 15 factors. Based on the fact that AKMV is highly similar to CPXV, mapping CPXV host range factors to AKMV may allow for speculation about the AKMV host range. The results demonstrated that the AKMV genome contains 26 host range factors that cover all 12 categories, a profile similar to CPXV_BR, implying that AKMV may be able to infect a wide range of animals similar to CPXV. The initial report detailing the isolation and identification of AKMV supports this notion, as AKMV infection was noted in humans and possibly cows. Furthermore, serological evidence suggests AKMV may also circulate in the rodent population [25]. However, 15 out of the 26 AKMV host range factors showed notable variation from those of CPXV_BR, indicating the AKMV optimum host might be different from that of CPXV. Notable limitations of this approach are that factors were analyzed outside of the genome context and that functional changes invoked by small residue changes were not considered [35]. Therefore, the predicted host range will need verification using animal experiments and/or ecological studies.

A recombination event was detected in the AKMV_VANI10 genome that suggests coinfection with AKMV and CPXV. For this to occur, circulating AKMV and CPXV must have overlapped geographically, and this may not have been a recent event. Considering the known wide host range of CPXV and the potential wide host range of AKMV, a phenomenon such as coinfection is not unexpected. Recombination between strains or species of OPXVs has been described previously [34,62,63]. The exchanged sequence in AKMV_VANI10 was about six kb, a size closely resembling the 3.5 kb size reported previously [34]. The recombined region of AKMV_VANI10 shares only 84.96% identity with AKMV_AKHM13, a 13% decrease compared with its identity to AKMV_AKHM13 at the left (97.80%) and right (98.29%) flanking regions. This supports the idea of the recombination region coming from a foreign origin. In contrast, the identity of the recombined region to CPXV is 9% higher than the flanking regions of each side. The recombined region included host range factors in the C7L and a KilA-N/RING domain-containing families, three of which exhibited positive selection in the OPXV lineage. Recombination resulted in unique, truncated versions of two proteins as well as a small deletion in a region under selection in a KilA-N/RING domain-containing protein, making the AKMV_VANI10 homolog more similar to the homologs in Old World OPXVs, such as CPXV. Such changes in proteins with predicted host range and virulence functions may suggest differences in the host range or virulence between the sequenced isolates. Future studies aimed at better understanding the diversity, ecology, and evolution of AKMV and other circulating OPXVs may help address how frequent or advantageous such a recombination event may be.

The AKMV genome sequence provides valuable information for future AKMV and OPXV research, for instance, in the development of nucleic acid diagnostic tools. During the initial typing of AKMV using a species-specific polymerase chain reaction (PCR) assay, a false positive result was observed in a MPXV-specific assay [25]. With the genome sequence available, the specificity of this assay can be improved. Similarly, the genome sequence would be helpful for developing species-specific serological tests by identifying unique antigen epitopes present in AKMV. Such an assay would answer the previous questions raised during the epidemiological investigation of AKMV infection [25]. In addition, AKMV genome sequences would facilitate research in other areas, such as therapeutic drug design, vaccine development, virus-host interaction characterization, and virulence factor studies.

## Supplementary Materials

The following are available online at http://www.mdpi.com/1999-4915/10/5/252/s1, Table S1. Strains used in this study; Table S2. Comparison of annotated genes in the ITR and terminal regions of AKMV_AKHM13-88, CXPV_BR, CPXV_GRI-90, and ECTV_MOS; Table S3. Comparison of annotated genes in the highly conserved central region of AKMV_AKHM13-88, CXPV_BR, CPXV_GRI-90, and ECTV_MOS; Table S4. The non-repetitive (NR) and tandem repeat (TR) regions in the ITR of Old World OPXVs; Table S5. Identity matrix of the highly conserved central region of the 22 selected orthopoxviruses (OPXVs); Table S6. Different genes between AKMV_AKMV13-88 and CPXV-BR in the ITR and terminal regions; Figure S1. Protein sequence alignment of AKMV009 with three similar proteins; Table S7. Comparison of the putative AKMV_AKHM13-88 host range factors to CPXV_BR; Table S8. Comparison of AKMV_VANI10 orthologs to the proteins of AKMV_AKHM13-88 and CPXV_GRI-90 encoded within or flanking the recombination region; Table S9. dN/dS analysis of proteins within or adjacent to the recombination region; Figure S2. Amino acid alignment of CPXV_GRI90 C3L homologs from representative OPXVs; and Figure S3. Amino acid alignment of KilA-N/RING domain containing proteins from representative OPXVs.
