# Supplementary material for: Genome Sequences of Akhmeta Virus, an Early Divergent Old World Orthopoxvirus
Source: Viruses. 2018 May 12;10(5):252. doi: 10.3390/v10050252 (PMC5977245; doi:10.3390/v10050252)
Supplement: Supplementary file 1 [file Figure S1_updated.pdf › Figure S1_updated.pdf]

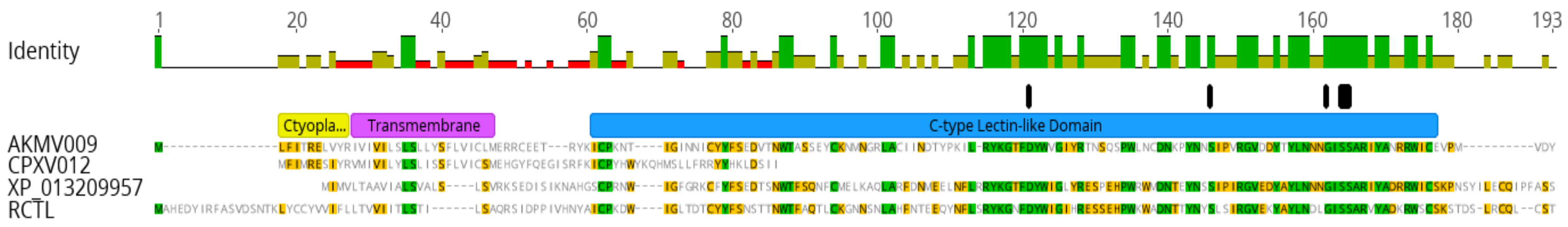

Figure S1. Protein sequence alignment of AKMV009 with three similar proteins: AKMV009 from AKMV_AKHM13-88, CPXV012 from CPXV_BR, XP_013209957 (a C-type lectin-like protein) from Microtus ochrogaster (Prairie Vole), and RCTL, the lectin-like molecule from cytomegalovirus. Sequence identity is displayed on the top (100% green, at least 30% and under 100% greeny-brown, under 30% red). Yellow box shows the predicted cytoplasmic domain; pink box indicates the transmembrance region; blue box shows the C-type lectin-like domain, and black boxes indicate the five residues essential for lectin binding to its ligand. –, indicate gaps in the alignment.
