# Supplementary material for: Genome Sequences of Akhmeta Virus, an Early Divergent Old World Orthopoxvirus
Source: Viruses. 2018 May 12;10(5):252. doi: 10.3390/v10050252 (PMC5977245; doi:10.3390/v10050252)
Supplement: Supplementary file 1 [file viruses-10-00252-s001.zip › Table S2.pdf]

Table S2. Comparison of annotated genes in the ITR and terminal regions of AKMV_AKHM13-88, CXPV_BR, CPXV_GRI-90, and ECTV_MOS

AKMV_AKHM13-88			CPXV_BR			CPXV_GRI-90			ECTV_MOS			Function
ORF	Position	Size	ORF	Size	Id %	ORF	Size	Id %	ORF	Size	Id%	
ITR region												
-			CPXV001			-			-			-
-			CPXV002			-			-			-
AKMV001	1,923-2,675	251	CPXV003	247	77	D1L	255	83	EVM001	248	81	Chemokine binding protein
-			CPXV004	72		-			-			-
AKMV002	2,794-3,840	349	CPXV005	356	83	D2L	351	88	-			TNF receptor
AKMV003	3,927-5,705	593	CPXV006	620	83	D3L	586	87	EVM002	588	88	Ankyrin
AKMV004	5,708-5,842	45	CPXV007	51	51	-			-			-
AKMV005	5,961-7,976	672	CPXV008	673	86	D4L	672	86	-			Ankyrin
AKMV006	8,161-8,622	154	CPXV009	154	92	D5L	153	94	-			-
-			-			-			EVM003	321		CrmD
Left terminal region												
-			CPXV010	216		D6L	219		-			Alpha-amanitin sensitivity protein
AKMV007	9,215-10,036	274	-			D7L	274	91	EVM004	273	90	Kelch-like protein
AKMV008	10,119-12,098	660	CPXV011	659	89	D8L	662	90	EVM005	651	88	Ankyrin
AKMV009	12,131-12,610	154	CPXV012	69	54	D10L	97	86	EVM007	104	84	C-type Lectin, host immunity evasion
-			-			D9L	76		EVM006	76		-
-			CPXV013	524		D11L	522		-			Kelch like
AKMV010	12,752-13,360	203	CPXV014	203	90	D12L	203	91	EVM008	203	88	TNF receptor
AKMV011	13,357-13,692	112	CPXV015	111	88	D13L	112	84	EVM009	112	84	-
AKMV012	13,800-16,100	767	CPXV016	765	80	D14L	765	80	EVM010	764	80	Ankyrin
AKMV013	16,497-17,783	429	CPXV017	436	78	C1L	438	80	-			Ankyrin
AKMV014	17,906-18,439	178	CPXV018	179	58	C2L	179	79	-			-
AKMV015	18,547-20,952	802	CPXV019	797	71	C3L	834	67	-			Ankyrin
AKMV016	21,060-21,566	169	CPXV020	171	73	C4L	171	75	-			-
AKMV017	21,741-22,154	138	CPXV021	140	78	C5R	139	77	-			Similar to viral growth factor
AKMV018	22,290-23,288	333	CPXV022	332	84	C6L	332	86	EVM011	332	85	IL-1 receptor antagonist
AKMV019	23,830-24,573	248	CPXV023	243	86	C7R	243	86	EVM012	242	82	Ubiquitin ligase
AKMV020	24,743-25,144	134	CPXV024	127	79	C8L	125	89	EVM013	139	79	IL-18 binding protein
AKMV021	25,175-27,193	673	CPXV025	669	79	C9L	669	80	-			Ankyrin
AKMV022	27,347-27,538	64	CPXV026	64	90	C10L	63	92	EVM014	60	94	-
AKMV023	27,718-29,652	645	CPXV027	633	84	C11L	615	65	-			Ankyrin
AKMV024	29,693-30,247	185	CPXV028	186	93	C12L	183	93	-			-
AKMV025	30,320-30,772	151	CPXV029	151	94	C13L	151	93	EVM015	151	91	Host-range factor
AKMV026	30,990-31,481	164	CPXV030	156	84	C14L	157	83	EVM016	156	85	-
AKMV027	31,641-31,781	47	CPXV031	70	69	-			-			Kelch-like protein
AKMV028	31,799-32,251	151	CPXV032	126	92	C15L	206	90	-			Kelch-like protein
AKMV029	32,328-33,269	314	CPXV033	317	82	C16L	316	80	-			IL-1 receptor antagonist
AKMV030	33,337-34,128	264	CPXV034	264	84	C17L	260	90	EVM017	263	88	Complement-binding protein
AKMV031	34,185-35,723	513	CPXV035	513	94	C18L	513	94	EVM018	513	92	Kelch-like protein
AKMV032	35,790-36,488	233	CPXV036	232	95	C19L	232	96	-			Putative TLR signaling inhibitor
AKMV033	36,466-36,819	118	CPXV037	118	84	Q1L	118	90	EVM019	118	86	NF-κB inhibitor
AKMV034	36,948-37,481	178	CPXV038	178	86	Q2L	176	91	EVM020	178	89	Alpha amanitin-sensitive protein
AKMV035	37,523-38,863	447	CPXV039	474	94	P1L	475	96	EVM021	473	96	Ankyin-like protein
AKMV036	38,908-39,570	221	CPXV040	221	96	P2L	164	95	-			NF-κB inhibitor
AKMV037	39,702-40,556	285	CPXV041	285	92	M1L	285	93	EVM022	286	93	Ankyrin-like protein
AKMV038	40,746-41,867	374	CPXV042	374	96	M2L	374	95	EVM023	374	93	Serpin
AKMV039	41,917-42,183	89	CPXV043	89	91	M3L	89	86	-			IFN resistance

AKMV040	42,244-43,518	425	CPXV044	425	97	M4L	425	97	-			Nicking/joining enzyme
AKMV041	43,545-44,378	278	CPXV045	277	97	M5L	277	98	EVM024	278	97	Monoglyceride lipase
AKMV042	44,518-44,967	150	CPXV046	150	96	M6R	162	94	-			Host immune response repressor
AKMV043	44,997-45,164	56	CPXV047	57	71	-			-			-
AKMV044	45,038-45,772	245	CPXV048	252	85	G1L	239	84	EVM025	457	83	Caspase-9 inhibitor
AKMV045	45,772-46,206	145	CPXV049	148	99	G2L	148	99	EVM026	148	99	dUTPase
AKMV046	46,240-47,682	481	CPXV050	481	94	G3L	486	95	EVM027	483	94	Kelch-like protein
AKMV047	47,693-48,652	320	CPXV051	334	97	G4L	320	98	EVM028	320	98	Ribonuclease reductase S subunit
AKMV048	48,685-49,653	323	CPXV052	324	87	G5L	324	90	-			Major Membrane protein
AKMV049	49,683-49,904	74	CPXV053	72	77	G6L	75	77	EVM029	75	77	-
AKMV050	49,920-50,159	80	CPXV054	82	79	G7L	81	86	EVM030	83	90	-
AKMV051	50,320-50,517	198	CPXV055	198	97	G8L	198	97	EVM031	198	95	-
Right terminal region												
AKMV156	149,726-153,367	1,214	CPXV158	1,284	72	A26L	1,279	72	EVM128	1,113	68	ATI protein
AKMV157	153,413-154,972	519	CPXV159	192	87	A27L	519	88	-			Fragment of P4c
-			CPXV160	69		-			-			-
-			CPXV161	261		-			-			Fragment of p4c
AKMV158	155,024-155,356	111	CPXV162	111	96	A28L	111	97	EVM129	111	95	IMV surface protein
AKMV159	155,357-155,797	147	CPXV163	147	97	A29L	147	99	EVM130	147	97	IMV membrane protein
AKMV160	155,798-156,715	306	CPXV164	306	99	A30L	306	99	EVM131	306	98	RNA Pol RPO35 protein
AKMV161	156,678-156,911	78	CPXV165	77	96	A31L	78	99	EVM132	78	97	IMV protein
AKMV162	157,071-157,454	128	CPXV166	141	84	A32R	146	94	EVM133	127	92	Hypothetical protein
AKMV163	157,424-158,185	254	CPXV167	312	100	A33L	301	100	EVM134	270	100	ATPase
AKMV164	158,366-158,920	185	CPXV168	188	94	A34R	186	95	EVM135	186	88	EEV membrane phosphoglycoprotein
AKMV165	158,944-159,450	169	CPXV169	169	99	A35R	169	99	EVM136	169	96	IEV and EEV membrane glycoprotein
AKMV166	159,431-159,610	60	CPXV170	62	93	-			-			-
AKMV167	159,495-160,025	177	CPXV171	177	99	A36R	177	98	EVM137	177	96	Hypothetical protein
AKMV168	160,116-160,760	215	CPXV172	225	93	A37R	224	95	-			IEV transmembrane phosphoprotein
AKMV169	160,825-161,616	264	CPXV173	264	95	A38R	269	95	-			Hypothetical protein
AKMV170	161,749-161,913	55	CPXV174	64	91	A39R	65	93	-			Hypothetical protein
AKMV171	161,910-162,743	278	CPXV175	278	97	A40L	278	96	EVM138	278	95	CD47-lilke protein
AKMV172	162,759-163,955	399	CPXV176	410	93	A41R	403	94	EVM139	400	94	Semaphorin
AKMV173	163,982-164,503	174	CPXV177	161	81	A42R	166	81	-			Secreted glycoprotein
AKMV174	164,618-165,280	221	CPXV178	219	77	A43L	220	78	EVM140	224	76	-
AKMV175	165,462-165,863	134	CPXV179	134	85	A44R	134	87	EVM141	135	87	Profilin-like protein
AKMV176	165,907-166,515	203	CPXV180	195	70	A45R	197	69	EVM142	212	88	Putative Type-1 membrane glycoprotein
AKMV177	166,534-166,761	76	CPXV181	82	83	A46R	79	78	-			Hypothetical protein
AKMV178	166,886-167,920	345	CPXV182	346	94	A47L	347	95	EVM143	347	93	3β-Hydroxysteroid dehydrogenase
AKMV179	167,968-168,345	126	CPXV183	126	96	A48R	126	97	EVM144	126	97	Cu-Zn superoxide dismutase-like protein
AKMV180	168,335-169,057	241	CPXV184	243	93	A49R	241	95	EVM145	241	95	TIR like protein
AKMV181	169,158-169,892	245	CPXV185	245	94	A50L	245	96	EVM146	245	94	Hypothetical protein
AKMV182	169,991-170,605	205	CPXV186	228	98	A51R	228	97	EVM147	228	96	Thymidylate kinase
AKMV183	170,654-171,142	163	CPXV187	163	93	A52R	163	93	-			Putative phosphotransferase protein
AKMV184	171,174-172,838	555	CPXV188	555	97	A53R	553	96	EVM148	555	98	DNA ligase
AKMV185	172,905-173,909	335	CPXV189	335	95	A54R	335	96	EVM149	335	95	Hypothetical protein
AKMV186	173,979-174,563	195	CPXV190	191	87	A55R	191	86	-			TIR like protein
AKMV187	174,913-175,467	185	CPXV191	187	77	A56R	187	76	-			Soluble TNF receptor CrmC
-			CPXV192	51		-			-			Ankyrin
AKMV188	175,708-177,402	565	CPXV193	564	94	A57R	565	94	EVM150	564	94	Kelch-like protein
AKMV189	177,452-178,393	314	CPXV194	298	81	A58R	315	86	EVM151	282	81	Haemagglutinin
AKMV190	178,410-179,003	198	CPXV195	198	99	A59R	198	99	-			Guanylate kinase
AKMV191	179,144-180,046	301	CPXV196	300	95	B1R	301	95	EVM152	300	94	Ser/Thr Protein kinase
AKMV192	180,139-181,653	505	CPXV197	506	82	B2R	504	84	EVM153	504	82	Schlafen-like protein

AKMV193	181,885-183,555	557	CPXV198	559	93	B3R	559	94	EVM154	565	93	Ankyrin
AKMV194	183,659-184,612	318	CPXV199	318	91	B4R	318	94	EVM155	318	93	EEV membrane glycoprotein
AKMV195	184,701-185,231	177	CPXV200	180	87	B5R	184	95	EVM156	178	98	Ankyrin
AKMV196	185,270-185,815	182	CPXV201	182	92	B6R	183	97	EVM157	182	99	Hypothetical protein
AKMV197	185,893-186,693	267	CPXV202	267	92	B7R	272	93	EVM158	267	99	Interferon-gamma receptor
AKMV198	186,835-187,581	249	CPXV203	226	84	B8R	222	66	-			Intracellular Viral protein
AKMV199	187,715-189,220	502	CPXV204	502	90	B9R	502	90	-			Kelch-like protein
AKMV200	189,291-189,578	96	CPXV205	91	93	B10R	106	96	EVM159	88	91	Hypothetical protein
AKMV201	189,645-190,502	286	CPXV206	286	95	B11R	284	94	EVM160	287	99	Ser/Thr Protein kinase
AKMV202	190,593-191,627	345	CPXV207	342	90	B12R	346	95	EVM161	345	96	Serpin
AKMV203	191,754-192,203	150	CPXV208	150	95	B13R	150	97	EVM162	165	100	Hypothetical protein
AKMV204	192,290-193,276	329	CPXV209	327	84	B14R	327	84	EVM163	329	98	IL-1β receptor
AKMV205	193,331-194,338	336	CPXV210	341	92	B15L	341	90	EVM164	341	96	Hypothetical protein
AKMV206	194,503-196,230	576	CPXV211	575	91	B16R	575	96	EVM165	595	95	Ankyrin
AKMV207	196,301-197,359	353	CPXV212	367	83	B17R	352	87	EVM166	359	84	IFN-α/β binding protein
AKMV208	197,440-199,833	798	CPXV213	801	87	B18R	796	91	-			Ankyrin
-			CPXV214	51		-			-			Unknown
AKMV209	199,940-201,619	560	CPXV215	558	78	B19R	558	79	EVM167	560	99	Kelch like
-			CPXV216	52		-			-			Unknown
AKMV210	201,927-203,009	361	CPXV217	373	89	B20R	376	89	EVM168	371	90	Serpin
AKMV211	203,213-203,431	72	CPXV218	197	70	B21R	197	72	-			Hypothetical protein
AKMV212	204,013-209,790	1,926	CPXV219	1,920	80	B22R	1,934	80	-			Surface Glycoprotein
AKMV213	210,043-211,785	581	CPXV220	580	94	K1R	582	97	-			-
-			CPXV221	322		-			-			CrmD
AKMV214	212,581-213,072	164	-			K3R	168	92	-			Receptor for host TNF/chemokines
-			-			T1R	211		-			-
ITR region												
-			-			-			EVM003	321		CrmD
AKMV215	213,302-213,763	154	CPXV222	154	92	I1R	153	94	-			-
AKMV216	213,948-215,963	672	CPXV223	675	86	I2R	672	86	-			-
AKMV217	216,082-216,216	45	CPXV224	51	51	-			-			-
AKMV218	216,228-217,997	590	CPXV225	620	83	I3R	586	87	EVM002	588	88	-
AKMV219	218,084-219,130	349	CPXV226	356	83	I4R	351	88	-			Soluble TNF receptor
AKMV220	219,249-220,001	251	CPXV227	247	77	I5R	255	83	EVM001	248	81	Chemokine binding protein
-			CPXV228	66		-			-			-
-			CPXV229	65		-			-			-

-, no gene annotation or unknown function; Id %, amino acid identity reported in Blast analysis; EEV, extracellular enveloped virion; IEV, intracellular enveloped virion; IMV, intracellular mature virion; IL, interleukin; NF-κB, nuclear factor kappa-light chain enhancer of activated B cells; TNF, tumor necrosis factor; TLR, toll-like receptor; IFN-γ, interferon gamma; Serpin, serine proteinase inhibitor.
