# Supplementary material for: Genome Sequences of Akhmeta Virus, an Early Divergent Old World Orthopoxvirus
Source: Viruses. 2018 May 12;10(5):252. doi: 10.3390/v10050252 (PMC5977245; doi:10.3390/v10050252)
Supplement: Supplementary file 1 [file viruses-10-00252-s001.zip › Table S3.pdf]

Table S3. Comparison of annotated genes in the highly conserved central region of AKMV_AKHM13-88, CXPV_BR, CPXV_GRI-90, and ECTV_MOS

AKMV_AKHM13-88			CPXV_BR			CPXV_GRI-90			ECTV_MOS			Functions
ORF	Position	Size	ORF	Size	Id %	ORF	Size	Id %	ORF	Size	Id%	
Conserved central region												
AKMV052	50,577-51,215	213	CPXV056	213	96	G9L	213	98	EVM032	213	98	S-S bond formation pathway protein
AKMV053	51,202-52,521	440	CPXV057	440	99	G10L	440	99	EVM033	440	99	Ser/Thr kinase
AKMV054	52,500-52,736	79	CPXV058	86	99	-			-			-
AKMV055	52,544-53,608	355	CPXV059	355	97	G11L	355	97	EVM034	355	96	-
AKMV056	53,667-55,577	637	CPXV060	635	95	G12L	635	95	EVM035	635	95	IEV maturation protein
AKMV057	55,610-56,728	373	CPXV061	373	98	G13L	373	98	EVM036	373	98	EEV phospholipase
AKMV058	56,746-56,973	76	CPXV062	74	80	G14L	74	69	EVM037	72	75	-
AKMV059	57,024-57,173	50	-			-			-			-
AKMV060	57,245-57,688	148	CPXV064	159	97	G15L	159	99	EVM038	159	99	-
AKMV061	57,728-58,423	232	CPXV065	232	94	G16L	232	95	EVM039	232	94	-
AKMV062	58,486-58,791	102	CPXV066	102	98	G17R	102	100	EVM040	102	97	DNA-binding virion core protein
AKMV063	58,788-60,227	480	CPXV067	480	98	F1L	480	99	EVM041	480	98	Poly(A) polymerase large subunit
AKMV064	60,224-62,437	738	CPXV068	738	98	F2L	738	98	EVM042	738	97	-
AKMV065	62,548-63,120	191	CPXV069	191	94	F3L	191	95	EVM043	191	93	Interferon resistance/PKR inhibitor
AKMV066	63,177-63,956	260	CPXV070	262	98	F4L	260	99	EVM044	260	99	RNA polymerase subunit
AKMV067	64,074-65,039	322	CPXV071	320	80	F5R	332	80	EVM045	332	80	-
AKMV068	65,251-66,954	568	CPXV072	568	96	F6R	568	95	EVM046	568	94	-
AKMV069	67,046-67,549	168	CPXV073	166	87	F7R	167	88	EVM047	166	87	Putative myristoylated protein
AKMV070	67,654-68,475	274	CPXV074	274	99	F8R	274	98	EVM048	274	97	-
AKMV071	68,481-71,513	1,011	CPXV075	1,006	98	F9L	1,007	97	EVM049	1,007	97	DNA polymerase
AKMV072	71,545-71,835	97	CPXV076	96	93	F10R	96	93	EVM050	96	92	Sulfhydryl oxidase
AKMV073	71,830-72,219	130	CPXV077	130	93	F11L	130	95	EVM051	130	90	Putative virion core protein
AKMV074	72,206-74,200	665	CPXV078	667	90	R1L	667	90	EVM052	667	90	-
AKMV075	74,216-74,404	63	CPXV078A	63	94	-			-			-
AKMV076	74,254-74,580	109	CPXV079	109	98	R2L	109	99	EVM053	109	97	Glutaredoxin
AKMV077	74,726-75,664	313	CPXV080	313	99	L1L	313	99	EVM054	313	98	Putative DNA-binding protein
AKMV078	75,671-75,895	75	CPXV081	74	92	L2L	74	93	EVM055	74	93	IMV membrane protein
AKMV079	75,896-76,705	270	CPXV082	269	97	L3L	270	97	EVM056	270	97	DNA-binding phosphoprotein
AKMV080	76,790-79,105	772	CPXV083	772	97	L4L	772	97	EVM057	772	97	Ribonucleotide reductase large subunit
AKMV081	79,133-79,372	80	CPXV084	80	98	L5L	80	99	EVM058	80	99	IMV protein
AKMV082	79,391-80,539	383	CPXV085	383	98	L6L	383	98	EVM059	383	97	Telomere binding protein
AKMV083	80,532-81,803	424	CPXV086	424	97	L7L	424	97	EVM060	424	97	Viral core cysteine proteinase
AKMV084	81,809-83,842	678	CPXV087	677	94	L8R	677	94	EVM061	677	95	RNA helicase NPH-II
AKMV085	83,846-85,576	577	CPXV088	592	97	H1L	592	97	EVM062	592	97	Putative metalloprotease
AKMV086	85,618-85,953	112	CPXV089	112	94	H2L	112	95	EVM063	112	94	Virus fusion complex
AKMV087	85,947-86,609	221	CPXV090	221	96	H3R	221	96	EVM064	221	95	Late Transcriptional factor
AKMV088	86,579-86,953	125	CPXV091	125	99	H4L	125	99	EVM065	125	100	Glutaredoxin-like protein
AKMV089	86,956-88,254	433	CPXV092	435	92	H5R	435	92	EVM066	435	92	-
AKMV090	88,262-88,453	64	CPXV093	64	100	H6R	64	100	EVM067	64	100	RNA polymerase

AKMV091	88,455-88,931	159	CPXV094	168	94	H7R	166	94	EVM068	166	94	NLPc/P60 superfamily protein
AKMV092	88,917-90,032	372	CPXV095	372	99	H8L	372	98	EVM069	372	98	Putative virion core protein
AKMV093	89,993-90,211	73	CPXV096	70	100	-			-			-
AKMV094	90,063-90,845	261	CPXV097	261	100	H9R	261	100	EVM070	261	100	Late transcription factor VLTF-1
AKMV095	90,873-91,895	341	CPXV098	341	95	H10R	341	95	EVM071	341	96	Entry fusion complex protein
AKMV096	91,896-92,648	251	CPXV099	251	99	N1R	251	100	EVM072	251	99	IMV membrane protein
AKMV097	92,680-92,943	88	CPXV100	87	90	N2R	93	93	EVM073	88	93	Cresent formation
AKMV098	92,933-93,994	354	CPXV101	351	95	N3L	351	95	EVM074	347	94	Internal virion protein
AKMV099	94,019-94,774	252	CPXV102	252	100	N4R	252	100	EVM075	252	99	DNA-binding virion core protein
AKMV100	94,784-95170	129	CPXV103	128	98	N5R	128	98	EMV076	128	98	IMV entry and fusion protein
AKMV101	95,154-95,588	145	CPXV104	153	98	S1R	154	98	EVM077	154	98	Virion protein
AKMV102	95,604-96,137	178	CPXV105	178	95	S2R	178	97	EVM078	178	96	Thymidine kinase
AKMV103	96,234-97,205	324	CPXV106	334	99	O1R	334	99	EVM079	334	99	Poly(A) polymerase small subunit
AKMV104	97,120-97,677	186	CPXV107	186	100	O2R	186	99	EVM080	186	99	Poly(A) polymerase small subunit
AKMV105	97,853-98,254	134	CPXV108	134	96	O3L	134	97	EVM081	134	96	membrane protein
AKMV106	98,360-102,220	1,287	CPXV109	1,287	99	O4R	1,287	99	EVM082	1,287	99	RNA polymerase subunit
AKMV107	102,217-102,732	172	CPXV110	172	99	J1L	172	100	EVM083	172	99	Try/Ser phosphatase
AKMV108	102,746-103,315	190	CPXV111	190	98	J2R	190	98	EVM084	190	97	Putative viral membrane protein
AKMV109	103,318-104,292	325	CPXV112	326	96	J3L	325	96	EVM085	325	94	Late transcription factor VLTF-4
AKMV110	104,293-106,680	796	CPXV113	796	98	J4L	796	99	EVM086	795	98	RNA polymerase-associated protein
AKMV111	106,866-107,492	209	CPXV114	207	92	J5R	213	96	EVM087	213	91	Late transcription factor 4 (VLTF-4)
AKMV112	107,493-108,437	315	CPXV115	315	97	J6R	315	97	EVM088	315	97	DNA topoisomerase Type I
AKMV113	108,393-108,593	67	CPXV116	61	92	-			-			-
AKMV114	108,481-108,915	145	CPXV117	147	94	J7R	147	94	EVM089	147	92	-
AKMV115	108,959-111,487	843	CPXV118	845	96	E1R	845	96	EVM090	844	96	mRNA capping enzyme large subunit
AKMV116	111,446-111,886	147	CPXV119	147	97	E2L	147	97	EVM091	147	95	Virion core protein
-			CPXV119A	80		-			-			-
AKMV117	111,879-112,592	238	CPXV120	238	97	E3R	238	97	EVM092	238	96	Virion core protein
AKMV118	112,592-113,248	219	CPXV121	219	98	E4R	219	98	EVM093	219	99	Uracil-DNA glycosylase
AKMV119	113,280-115,637	786	CPXV122	786	98	E5R	786	99	EVM094	786	98	NTPase
AKMV120	115,678-117,591	638	CPXV123	638	99	E6R	638	99	EVM095	638	98	VETF early transcription factor
AKMV121	117,618-118,103	162	CPXV124	162	97	E7R	162	98	EVM096	162	98	RNA polymerase subunit RPO18
AKMV122	118,066-118,980	305	CPXV125	305	94	E8L	305	94	EVM097	305	95	IMV membrane protein
AKMV123	119,022-119,663	214	CPXV126	214	99	E9R	214	99	EVM098	214	99	mRNA decapping enzyme
AKMV124	119,660-120,406	249	CPXV127	249	100	E10R	249	99	EVM099	251	97	mRNA decapping enzyme
AKMV125	120,407-122,302	632	CPXV128	632	99	E11L	632	99	EVM100	632	98	ATPase
AKMV126	122,336-123,199	288	CPXV129	288	99	E12L	288	99	EVM101	288	99	mRNA capping enzyme small subunit
AKMV127	123,161-123,379	73	CPXV130	73	97	-			-			-
AKMV128	123,230-124,885	552	CPXV131	552	99	E13L	552	99	EVM102	552	99	Trimeric virion coat protein
AKMV129	124,909-125,361	151	CPXV132	151	98	A1L	151	97	EVM103	151	97	late transcription factor VLTF-2
AKMV130	125,382-126,056	225	CPXV133	225	100	A2L	225	99	EVM104	225	99	Late transcription factor VLTF-3
AKMV131	126,053-126,283	77	CPXV134	77	84	A3L	77	86	EVM105	77	83	S-S bond formation pathway protein
AKMV132	126,298-128,232	645	CPXV135	645	97	A4L	645	98	EVM106	645	98	P4b precursor
AKMV133	128,285-129,133	283	CPXV136	296	86	A5L	284	92	EVM107	282	92	Core protein

AKMV134	129,171-129,665	165	CPXV137	165	99	A6R	165	99	EVM108	165	98	RNA polymerase
AKMV135	129,662-130,780	373	CPXV138	373	97	A7L	373	97	EVM109	373	96	Virion core and cleavage processing protein
AKMV136	130,804-132,936	711	CPXV139	711	99	A8L	711	99	EVM110	711	98	Early gene transcription factor
AKMV137	132,990-133,856	289	CPXV140	289	99	A9R	289	99	EVM111	289	99	VITF-3 32kd small subunit
AKMV138	133,893-134,195	101	CPXV141	122	96	A10L	108	97	EVM112	111	97	Early morphogenesis protein
AKMV139	134,196-136,874	893	CPXV142	895	95	A11L	892	97	EVM113	892	97	P4a precursor
AKMV140	136,889-137,845	319	CPXV143	319	100	A12R	319	100	EVM114	319	99	Virial membrane formation
AKMV141	137,847-138,419	191	CPXV144	191	97	A13L	195	93	EVM115	192	94	Virion core and cleavage processing protein
AKMV142	138,443-138,661	73	CPXV145	71	90	A14L	69	88	EVM116	67	86	IMV membrane protein
AKMV143	138,768-139,040	91	CPXV146	91	99	A15L	91	100	EVM117	91	100	Phosphorylated IMV membrane protein
AKMV144	139,057-139,218	54	CPXV147	54	100	-			EVM117.5	54	100	Hydrophobic IMV membrane protein
AKMV145	139,208-139,492	95	CPXV148	95	98	A16L	95	98	EVM118	95	96	Core protein
AKMV146	139,476-140,609	378	CPXV149	378	97	A17L	378	96	EVM119	378	97	Myristylate protein for entry
AKMV147	140,612-141,235	208	CPXV150	203	95	A18L	204	95	EVM120	203	92	IMV membrane protein
AKMV148	141,250-142,734	495	CPXV151	493	96	A19R	494	95	EVM121	494	95	DNA helicase
AKMV149	142,715-142,948	78	CPXV152	78	99	A20L	77	99	EVM122	78	96	Zinc finger-like protein
AKMV150	142,918-143,046	43	CPXV152A	82	93	-			-			-
AKMV151	142,949-143,302	118	CPXV153	118	97	A21L	118	98	EVM123	119	96	IMV membrane protein
AKMV152	143,301-144,581	427	CPXV154	427	95	A22R	427	96	EVM124	427	95	DNA Polymerase processing factor
AKMV153	144,556-145,074	173	CPXV155	188	99	A23R	188	99	EVM125	188	98	Holliday junction endonuclease
AKMV154	145,094-146,242	383	CPXV156	383	98	A24R	383	98	EVM126	383	98	VITF-3 large subunit
AKMV155	146,239-149,733	1,165	CPXV157	1,165	99	A25R	1,165	99	EVM127	1,165	99	DNA-directed RNA polymerase

-, no gene annotation or unknown function; Id %, amino acid identity from local BLAST; EEV, extracellular enveloped virion; IEV, intracellular enveloped virion; IMV, intracellular mature virion; VITF, vaccinia virus intermediate transcription factors.
