# Supplementary material for: Genome Sequences of Akhmeta Virus, an Early Divergent Old World Orthopoxvirus
Source: Viruses. 2018 May 12;10(5):252. doi: 10.3390/v10050252 (PMC5977245; doi:10.3390/v10050252)
Supplement: Supplementary file 1 [file viruses-10-00252-s001.zip › Table S5.pdf]

Table S5. Identity matrix of the highly conserved central region of the 22 selected OPXVs

	1	2	3	4	5	6	7	8	9	10	11	12	13	14	15	16	17	18	19	20	21	22
1. AKMV_AKHM13-88																						
2. AKMV_VANI10	99.42																					
3. CPXV_AUS99	95.76	95.71																				
4. CPXV_GRI-90	95.71	95.67	98.87																			
5. CPXV_HumLit08/1	95.72	95.69	98.96	98.95																		
6. CPXV_HumGra07/1	95.81	95.76	98.73	98.51	98.69																	
7. CPXV_FRA01	95.42	95.38	97.90	98.03	98.01	97.83																
8. CPXV_BR	95.42	95.39	97.82	97.97	97.93	97.75	98.67															
9. CPXV_GER02	95.74	95.70	98.47	98.42	98.53	98.55	98.18	98.07														
10. TATV_DAH	95.68	95.64	98.46	98.28	98.52	98.95	97.65	97.53	98.32													
11. VACV_LIST107	95.57	95.54	98.80	98.74	98.82	98.42	97.79	97.73	98.36	98.21												
12. VACV_CVA	95.57	95.52	98.78	98.73	98.87	98.45	97.81	97.77	98.37	98.23	99.42											
13. VACV_ACAM	95.54	95.50	98.77	98.72	98.82	98.42	97.79	97.73	98.33	98.20	99.37	99.46										
14. RPXV_UTR	95.52	95.49	98.76	98.73	98.83	98.38	97.79	97.74	98.33	98.17	99.39	99.40	99.33									
15. CMLV_CMS	95.48	95.44	98.23	98.07	98.30	98.72	97.43	97.30	98.10	99.24	97.98	98.00	97.98	97.95								
16. ECTV_MOS	95.37	95.35	97.59	97.67	97.61	97.49	97.43	97.34	97.49	97.24	97.42	97.41	97.43	97.41	97.02							
17. HSPV_MNR76	95.30	95.26	98.61	98.37	98.55	98.24	97.48	97.41	97.96	98.01	99.05	99.07	99.15	99.04	97.79	97.09						
18. MPXV_LIB70	95.15	95.11	98.17	98.19	98.18	97.87	97.49	97.43	97.84	97.71	98.00	98.02	98.01	98.06	97.49	97.15	97.70					
19. MPXV_ZAI96	95.12	95.10	98.16	98.19	98.17	97.86	97.48	97.41	97.84	97.69	98.01	98.02	98.01	98.06	97.48	97.14	97.69	99.66				
20. VARV_BSH74	95.13	95.10	97.78	97.62	97.83	98.26	97.02	96.90	97.65	98.75	97.55	97.57	97.56	97.53	98.54	96.66	97.37	97.10	97.07			
21. VARV_BRZ66	95.12	95.10	97.79	97.62	97.84	98.27	97.03	96.92	97.66	98.76	97.56	97.58	97.57	97.54	98.54	96.65	97.38	97.10	97.08	99.72		
22. RCNV_HRM	86.73	86.72	86.79	86.85	86.84	86.82	86.77	86.82	86.79	86.75	86.72	86.74	86.72	86.73	86.65	86.65	86.62	86.58	86.56	86.51	86.52	
