# Supplementary material for: Genome Sequences of Akhmeta Virus, an Early Divergent Old World Orthopoxvirus
Source: Viruses. 2018 May 12;10(5):252. doi: 10.3390/v10050252 (PMC5977245; doi:10.3390/v10050252)
Supplement: Supplementary file 1 [file viruses-10-00252-s001.zip › Table S7.pdf]

Table S7. Comparison of the putative AKMV_AKHM13-88 host range factors to CPXV_BR

Category	Host range factor	Gene in AKMV	Homolog in CPXV_BR	Accession #	Size comparison AKMV/CPXV_BR	Amino Acid Identity %
-	K3L	*AKMV039*	*CPXV043*	NP_619832	88/88	90.91
-	E3L	*AKMV065*	*CPXV069*	NP_619856	190/190	93.68
C7L/C4	C7L	*AKMV025*	*CPXV029*	NP_619818	150/150	94.00
	C4L	*AKMV016**	*CPXV020*	NP_619809	168/170	72.94
TNFR II Family	CrmB	*AKMV002**	*CPXV005*	NP_619793	348/355	82.91
	CrmC	*AKMV187**	*CPXV191*	NP_619973	168/187	76.92
	CrmD	-*	*CPXV221*	NP_620001		
	CrmE	*AKMV217**	-			
-	T4	*AKMV198**	*CPXV203*	NP_619984	249/226	84.44
Bcl-2 family	M11/F1L	*AKMV044**	*CPXV048*	NP_619836	244/251	84.52
Serpins	SPI-1	*AKMV210**	*CPXV217*	NP_619997	360/372	88.61
	SPI-2	*AKMV202*	*CPXV207*	NP_619988	344/341	90.99
	SPI-3	*AKMV038*	*CPXV042*	NP_619831	373/373	95.71
-	P28-like	*AKMV019**	*CPXV023*	NP_619812	247/242	86.69
-	B5R	*AKMV194*	*CPXV199*	NP_619980	317/317	91.80
-	C3L	*AKMV030**	*CPXV034*	NP_619823	263/263	84.03
ANK/F-box	Group 1	*AKMV208**	*CPXV213*	NP_619994	797/800	87.27
	Group 2	*AKMV008*	*CPXV011*	NP_619800	659/658	90.24
	Group 3	*AKMV206*	*CPXV211*	NP_619992	575/574	90.96
	Group 4	*AKMV212*	*CPXV220*	NP_620000	580/579	93.80
	Group 5	*AKMV003**	*CPXV006*	NP_619794	592/619	82.44
	Group 6	*AKMV193*	*CPXV198*	NP_619979	556/558	93.19
	Group 7	*AKMV021**	*CPXV025*	NP_619814	672/668	79.02
	Group 8	*AKMV013**	*CPXV017*	NP_619806	428/435	78.37
	Group 9	*AKMV023**	*CPXV027*	NP_619816	644/632	83.72
	Group 10	*AKMV005**	*CPXV008*	NP_619796	671/672	85.58
-	K1L	*AKMV037*	*CPXV041*	NP_619830	284/284	92.25

*, factors showing lower identity (<90%), or deletion, or truncation between AKMV_AKHM13-88 and CPXV-BR.
