# Supplementary material for: Genome Sequences of Akhmeta Virus, an Early Divergent Old World Orthopoxvirus
Source: Viruses. 2018 May 12;10(5):252. doi: 10.3390/v10050252 (PMC5977245; doi:10.3390/v10050252)
Supplement: Supplementary file 1 [file viruses-10-00252-s001.zip › Table S8.pdf]

Table S8. Comparison of AKMV_VANI10 orthologs to the proteins of AKMV_AKHM13-88 and CPXV_GRI-90 encoded within or flanking the recombination region

AKMV_VANI10 (tentative)	AKMV_AKHM13	CPXV_GRI-90
ORF013	**99.53** (AKMV013)	**79.32** (C1L)
ORF014	**80.34** (AKMV014)	**73.60** (C2L)
ORF015*	**74.29** (AKMV015)	**90.43** (C3L)
ORF016	**73.84** (AKMV016)	**89.53** (C4L)
ORF017	**76.26** (AKMV017)	**86.96** (C5R)
ORF018	**84.73** (AKMV018)	**94.26** (C6L)
OFF019	**85.08** (AKMV019)	**94.21** (C7R)
ORF020	**86.05** (AKMV020)	**90.70** (C8L)
ORF021	**97.62** (AKMV021)	**79.70** (C9L)

Bold number is aa identity; gene names are indicated in parenthesis; *, identity after a 281 aa internal gap was stripped from protein sequence alignment.
