# Supplementary material for: Genome Sequences of Akhmeta Virus, an Early Divergent Old World Orthopoxvirus
Source: Viruses. 2018 May 12;10(5):252. doi: 10.3390/v10050252 (PMC5977245; doi:10.3390/v10050252)
Supplement: Supplementary file 1 [file viruses-10-00252-s001.zip › Table S6.pdf]

Table S6. Different genes between AKMV_AKMV13-88 and CPXV-BR in the ITR and terminal regions

AKMV	CPXV_BR	Function	Reference
1. Different presence			
-	*CPXV001*	Integral component of membrane	-
-	*CPXV002*	-	-
-	*CPXV004*	Integral component of membrane	-
-	*CPXV010*	-	-
-	*CPXV013*	Kelch-like protein	[1]
-	*CPXV160*	Integral component of membrane	-
-	*CPXV161*	A29 homolog, virus budding	-
-	*CPXV192*	Integral component of membrane	-
-	*CPXV214*	-	-
-	*CPXV216*	S signal peptide	-
-	*CPXV221*	Tumor necrosis factor receptor	[2, 3]
AKMV007	-	Kelch repeat and BTB-containing domain	-
AKMV214	-	Receptor of TNF and various chemokines	[2]
2. Truncated in AKMV			
AKMV004	*CPXV007*	-	-
AKMV156	*CPXV158*	ATI protein	-
AKMV162	*CPXV166*	Hypothetical protein	-
AKMV163	*CPXV167*	DNA packaging	-
AKMV182	*CPXV186*	Thymidylate kinase	-
AKMV211	*CPXV218*	Chemokine binding	-
3. Truncated in CPXV			
AKMV009	*CPXV012*	Similar to Clr-B, inhibits NK cell function	[4]
AKMV157	*CPXV159*	Prevents virus-cell direct fusion with ATI	
AKMV198	*CPXV203*	Disrupts MHCI trafficking to cell surface	[5, 6]
4. Fragmented differently			
AKMV027	*CPXV031*	-	-
AKMV028	*CPXV032*	Similar to C5L, Kelch-containing protein	-
AKMV200	*CPXV205*	B11 family	-

References

1. Kochneva, G.; Kolosova, I.; Maksyutova, T.; Ryabchikova, E.; Shchelkunov, S., Effects of deletions of kelch-like genes on cowpox virus biological properties. *Archives of virology* **2005,** 150, (9), 1857-70.
2. Alejo, A.; Ruiz-Arguello, M. B.; Ho, Y.; Smith, V. P.; Saraiva, M.; Alcami, A., A chemokine-binding domain in the tumor necrosis factor receptor from variola (smallpox) virus. *Proceedings of the National Academy of Sciences of the United States of America* **2006,** 103, (15), 5995-6000.
3. Loparev, V. N.; Parsons, J. M.; Knight, J. C.; Panus, J. F.; Ray, C. A.; Buller, R. M.; Pickup, D. J.; Esposito, J. J., A third distinct tumor necrosis factor receptor of orthopoxviruses. *Proceedings of the National Academy of Sciences of the United States of America* **1998,** 95, (7), 3786-91.
4. Luteijn, R. D.; Hoelen, H.; Kruse, E.; van Leeuwen, W. F.; Grootens, J.; Horst, D.; Koorengevel, M.; Drijfhout, J. W.; Kremmer, E.; Fruh, K.; Neefjes, J. J.; Killian, A.; Lebbink, R. J.; Ressing, M. E.; Wiertz, E. J., Cowpox virus protein CPXV012 eludes CTLs by blocking ATP binding to TAP. *Journal of immunology* **2014,** 193, (4), 1578-89.
5. McCoy, W. H. t.; Wang, X.; Yokoyama, W. M.; Hansen, T. H.; Fremont, D. H., Structural mechanism of ER retrieval of MHC class I by cowpox. *PLoS biology* **2012,** 10, (11), e1001432.
6. McCoy, W. H. t.; Wang, X.; Yokoyama, W. M.; Hansen, T. H.; Fremont, D. H., Cowpox virus employs a two-pronged strategy to outflank MHCI antigen presentation. *Mol Immunol* **2013,** 55, (2), 156-8.
