# Supplementary material for: Genome Sequences of Akhmeta Virus, an Early Divergent Old World Orthopoxvirus
Source: Viruses. 2018 May 12;10(5):252. doi: 10.3390/v10050252 (PMC5977245; doi:10.3390/v10050252)
Supplement: Supplementary file 1 [file viruses-10-00252-s001.zip › Table S9.pdf]

Table S9. dN/dS analysis of proteins within or adjacent to the recombination region

CPXV_BR	CPXV_ GRI-90	AKMV	Predicted Function	Lrt Statistic (p value)	Sites: AKMV_AKHM13	Sites: AKMV_VANI10	Homologs Compared (reference genomes)
CPXV020	C4L	AKMV016	Host range defense	15.40<br>p<0.001	158N	162N	CPXV_BR, CPXV_GRI-90, CPXV_AUS99, CPXV_HUMGRA07/1, CPXV_HUMLIT08/1, CPXV_FRA01, CPXV_GER91, CPXV_GER02, CMLV_CMS, SKPV_WA, VPXV_CA
CPXV021	C5R	AKMV017	Secreted EGF-like protein, viral growth factor VGF	0.04<br>NS	-	-	CPXV_BR, CPXV_GRI-90, CPXV_AUS99, CPXV_HUMGRA07/1, CPXV_HUMLIT08/1, CPXV_FRA01, CPXV_GER91, CPXV_GER02, HSPV_NMR76, CMLV_CMS, TATV_DAH, RPXV_UTR, MPXV_ZAI96, MPXV_LIB70, VARV_BRZ66, SKPV_WA, VPXV_CA
CPXV022	C6L	AKMV018	IL-1 receptor antagonist	1.19<br>NS	-	-	CPXV_BR, CPXV_GRI-90, CPXV_AUS99, CPXV_HUMGRA07/1, CPXV_HUMLIT08/1, CPXV_FRA01, CPXV_GER91, CPXV_GER02, HSPV_NMR76, ECTV_MOS, RPXV_UTR, VARV_BRZ66
CPXV023	C7R	AKMV019	p28/N1R family, KilA-N RING ubiquitin ligase	37.48<br>p<0.001	133T<br>142R<br>224I	134A<br>137H<br>219S	CPXV_BR, CPXV_GRI-90, CPXV_AUS99, CPXV_HUMGRA07/1, CPXV_HUMLIT08/1, CPXV_FRA01, CPXV_GER91, CPXV_GER02, ECTV_MOS, CMLV_CMS, TATV_DAH, RPXV_UTR, MPXV_ZAI96, MPXV_LIB70, VARV_BRZ66, SKPV_WA, VPXV_CA
CPXV024	C8L	AKMV020	IL-18 binding	0<br>NS	-	-	CPXV_BR, CPXV_GRI-90, CPXV_AUS99, CPXV_HUMGRA07/1, CPXV_HUMLIT08/1, CPXV_FRA01, CPXV_GER91, CPXV_GER02, HSPV_NMR76, ECTV_MOS, TATV_DAH, RPXV_UTR, MPXV_ZAI96, MPXV_LIB70, VARV_BRZ66, SKPV_WA
CPXV025	C9L	AKMV021	Ankyrin, host range VHRI	0<br>NS	-	-	CPXV_BR, CPXV_GRI-90, CPXV_AUS99, CPXV_HUMGRA07/1, CPXV_HUMLIT08/1, CPXV_FRA01, CPXV_GER91, CPXV_GER02, TATV_DAH, MPXV_ZAI96, MPXV_LIB70, SKPV_WA, VPXV_CA
CPXV029	C13L	AKMV025	Host range defense	9.49<br>p<0.01	38G	38G	CPXV_BR, CPXV_GRI-90, CPXV_AUS99, CPXV_HUMGRA07/1, CPXV_HUMLIT08/1, CPXV_FRA01, CPXV_GER91, CPXV_GER02, HSPV_NMR76, ECTV_MOS, CMLV_CMS, TATV_DAH, RPXV_UTR, MPXV_ZAI96, MPXV_LIB70, VARV_BRZ66, SKPV_WA, VPXV_CA
CPXV030	C14L	AKMV026	Unknown	1.15<br>NS	-	-	CPXV_BR, CPXV_GRI-90, CPXV_AUS99, CPXV_HUMGRA07/1, CPXV_HUMLIT08/1, CPXV_FRA01, CPXV_GER91, CPXV_GER02, HSPV_NMR76, ECTV_MOS, CMLV_CMS, TATV_DAH, RPXV_UTR, MPXV_ZAI96, MPXV_LIB70, VARV_BRZ66, SKPV_WA, VPXV_CA
