# Supplementary material for: Genome Sequences of Akhmeta Virus, an Early Divergent Old World Orthopoxvirus
Source: Viruses. 2018 May 12;10(5):252. doi: 10.3390/v10050252 (PMC5977245; doi:10.3390/v10050252)
Supplement: Supplementary file 1 [file viruses-10-00252-s001.zip › Table S4.pdf]

Table S4. The NR and TR regions in the ITR of Old World OPXVs

Genomes	NR1 (bp)	TR1 (bp)	Major Pattern of Repeat in TR1	NR2 (bp)	TR2 (bp)	Major Pattern of Repeat in TR2	NR3 (bp)	Full ITR (bp)^
VACV_WR	81	912	**70** (12.7)	306	2,036	**70** (19); **54** (8.3)	6,742	10,077
HSPV_MNR76	81	92	**70** (1.3)	304	108	**6** (3)	6,922	7,507
AKMV_AKHM13-88	78	604	**86** (6.3)	300	740	**60** (11.5)	7,003	8,725
CPXV_BR	81	1,515	**78** (2.7); **177** (7.5)	303	537	**125** (3.2)	7,165	9,600
CPXV_GRI-90	81	489	**70** (6.8)	309	291	**39** (2.2)	7,108	8,278
ECTV_ERPV	80	162	**70** (2.3)	304	937	**85** (10.4)	5,540	7,022
MPXV_CNG03	75	280	**70** (3.7)	300	104	**8** (2.2)	5,719	6,477
CMLV_CMS*	81	264	**71**(2.7)	n/a	102	**6** (10)	5,602	6,049
TATV_DAH	72#	141	**33** (3.5)$	303	138	**6** (3)	4,124	4,779
VARV_CHN48	75	345	**69** (4.8)	302	n/a	n/a	n/a	728

Bold number indicates the length of a repeat unit; copy number is in parenthesis; fraction indicates the presence of an incomplete repeat unit. *, lacking NR2; #, short NR1 probably due to insufficient sequencing at the genomic terminus; $, low percent match (68%); ^ the bases in the terminal hairpins are excluded.
