# Supplementary material for: Genome Sequences of Akhmeta Virus, an Early Divergent Old World Orthopoxvirus
Source: Viruses. 2018 May 12;10(5):252. doi: 10.3390/v10050252 (PMC5977245; doi:10.3390/v10050252)
Supplement: Supplementary file 1 [file viruses-10-00252-s001.zip › Figure S2_updated.pdf]

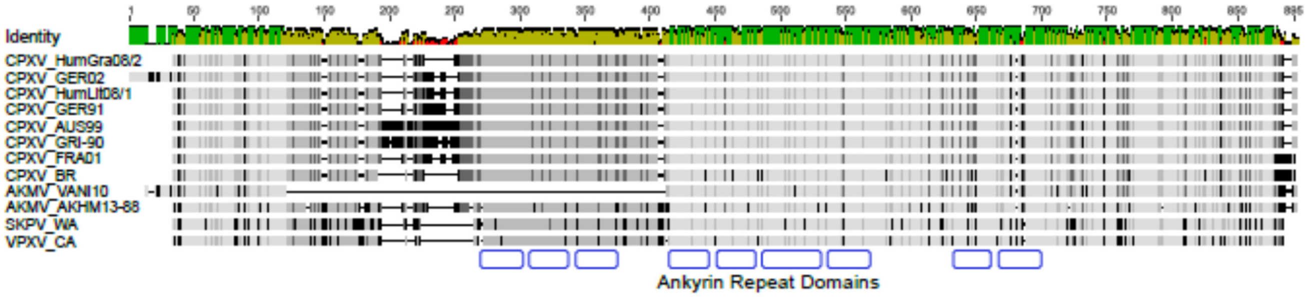

Figure S2. Amino acid alignment of CPXV_GRI-90 C3L homologs from representative OPXVs. AKMV_VANI10 exhibited a large deletion spanning three ankyrin repeat domains. Sequence identity and position are shown above (100% green, at least 30% and under 100% greeny-brown, under 30% red). Shaded bars indicate amino acid similarity based on BLOSUM62 (100% light gray, 80-100% medium gray, 60-80% dark gray, less than 60% black). Alignment gaps are shown by horizontal lines. Location of predicted ankyrin repeat domains are shown with blue boxes below.
