# Supplementary material for: Genome Sequences of Akhmeta Virus, an Early Divergent Old World Orthopoxvirus
Source: Viruses. 2018 May 12;10(5):252. doi: 10.3390/v10050252 (PMC5977245; doi:10.3390/v10050252)
Supplement: Supplementary file 1 [file viruses-10-00252-s001.zip › Figure S3_updated.pdf]

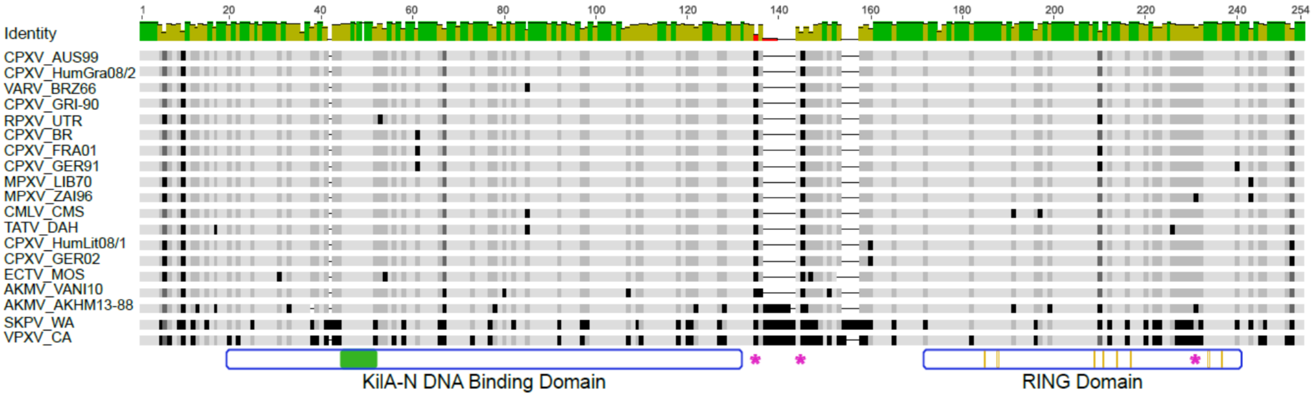


**Figure S3.** Amino acid alignment of KilA-N/RING domain containing proteins from representative OPXVs. AKMV_VANI10 exhibited a small deletion in a region flanked by two sites under positive selection (pink asterisks), rendering it more similar to Old World OPXVs. Sequence identity and position are shown above (100% green, at least 30% and under 100% greeny-brown, under 30% red). Shaded bars indicate amino acid similarity based on BLOSUM62 (100% light gray, 80-100% medium gray, 60-80% dark gray, less than 60% black). Alignment gaps are shown by horizontal lines. Location of predicted KilA-N DNA Binding and RING domains are shown with blue boxes below. DNA binding region within the KilA-N domain is highlighted by a green box. Conserved Cysteine residues important for ubiquitin ligase function of the RING domain are shown with yellow bars.
