# Supplementary material for: Genome Sequences of Akhmeta Virus, an Early Divergent Old World Orthopoxvirus
Source: Viruses. 2018 May 12;10(5):252. doi: 10.3390/v10050252 (PMC5977245; doi:10.3390/v10050252)
Supplement: Supplementary file 1 [file viruses-10-00252-s001.zip › Table S1_updated.pdf]

Table S1. Strains used in this study

Species	Strain	Abbreviation	Accession #	Size	Reference
Akhmeta virus	Akhmeta2013-85	AKMV_AKHM13-85		221,911	This study
Akhmeta virus	Akhmeta2013-88	AKMV_AKHM13-88		221,902	This study
Akhmeta virus	Vani2010	AkMV_VANI10		217,740	This study
Cowpox virus	GRI-90	CPXV_GRI-90	X94355.2	223,666	[1]
Cowpox virus	Brighton Red	CPXV_BR	NC_003660.2	224,499	[2]
Cowpox virus	Germany 91-3	CPXV_GER91	DQ437593.1	228,250	[3]
Cowpox virus	Austria 1999	CPXV_AUS99	HQ407377.1	221,512	[4]
Cowpox virus	HumGra07/1	CPXV_HumGra08/2	KC813510.1	220,071	[5]
Cowpox virus	RatKre08/2	CPXV_RatKre08/2	KC813505.1	221,194	[5]
Cowpox virus	JagKre08/1	CPXV_JagKre08/1	KC813497.1	216,415	[5]
Cowpox virus	HumMag07/1	CPXV_HumMag07/1	KC813495.1	217,406	[5]
Cowpox virus	HumLit08/1	CPXV_HumLit08/1	KC813493.1	215,715	[5]
Cowpox virus	France_2001_Nancy	CPXV_FRA01	HQ420894.1	226,424	[4]
Cowpox virus	Germany_2002_MKY	CPXV_GER02	HQ420898.1	222,555	[4]
Ectromelia virus	Moscow	ECTV_MOS	NC_004105.1	209,771	[6]
Ectromelia virus	ERPV	ECTV_ERPV	JQ410350.1	206,527	[7]
Ectromelia virus	Naval	ECTV_NAV	KJ563295.1	207,428	[8]
Taterapox virus	Dahomey 1968	TATV_DAH	NC_008291.1	198,050	[3]
Camelpox virus	CMS	CMLV_CMS	AY009089.1	202,205	[9]
Rabbitpox virus	Rabbitpox Utrecht	RPXV_UTR	AY484669.1	197,731	[10]
Horsepox virus	MNR-76	HSPV_NMR76	DQ792504	212,633	[11]
Monkeypox virus	Congo_2003_358	MPXV_CNG03	DQ011154.1	197,195	[12]
Monkeypox virus	Liberia_1970_184	MPXV_LIB70	DQ011156.1	200,256	[12]
Monkeypox virus	Zaire-96-I-16	MPXV_ZAI96	NC_003310.1	196,858	[12]
Vaccinia virus	Lister clone VACV107	VACV_LIST107	DQ121394.1	189,421	[13]
Vaccinia virus	Chorioallantois vaccinia virus Ankara	VACV_CVA	AM501482.1	192,353	[14]
Vaccinia virus	Acambis clone 2000	VACV_ACAM	AY313847.1	199,234	[15]
Vaccinia virus	Western Reserve	VACV_WR	NC_006998	194,711	[16]
Variola virus	Brazil 1966	VARV_BRZ66	DQ441419.1	188,062	[3]
Variola virus	Bangladesh 1974	VARV_BSH74	DQ441420.1	186,293	[3]
Variola virus	China Horn 1948	VARV_CHN48	DQ437582.1	186,668	[3]
Raccoonpox virus	Herman	RCNV_HRM	NC_027213.1	214,699	[17]
Skunkpox virus	WA	SKPV_WA	KU749310.1	222,832	[18]
Volepox virus	CA	VPXV_CA	KU749311.1	217,124	[18]

References

1. Shchelkunov, S. N.; Safronov, P. F.; Totmenin, A. V.; Petrov, N. A.; Ryazankina, O. I.; Gutorov, V. V.; Kotwal, G. J., The genomic sequence analysis of the left and right species-specific terminal region of a cowpox virus strain reveals unique sequences and a cluster of intact ORFs for immunomodulatory and host range proteins. *Virology* **1998,** 243, (2), 432-60.
2. Pickup, D. J.; Bastia, D.; Stone, H. O.; Joklik, W. K., Sequence of terminal regions of cowpox virus DNA: arrangement of repeated and unique sequence elements. *Proceedings of the National Academy of Sciences of the United States of America* **1982,** 79, (23), 7112-6.
3. Esposito, J. J.; Sammons, S. A.; Frace, A. M.; Osborne, J. D.; Olsen-Rasmussen, M.; Zhang, M.; Govil, D.; Damon, I. K.; Kline, R.; Laker, M.; Li, Y.; Smith, G. L.; Meyer, H.; Leduc, J. W.; Wohlhueter, R. M., Genome sequence diversity and clues to the evolution of variola (smallpox) virus. *Science* **2006,** 313, (5788), 807-12.
4. Carroll, D. S.; Emerson, G. L.; Li, Y.; Sammons, S.; Olson, V.; Frace, M.; Nakazawa, Y.; Czerny, C. P.; Tryland, M.; Kolodziejek, J.; Nowotny, N.; Olsen-Rasmussen, M.; Khristova, M.; Govil, D.; Karem, K.; Damon, I. K.; Meyer, H., Chasing Jenner's vaccine: revisiting cowpox virus classification. *PloS one* **2011,** 6, (8), e23086.
5. Dabrowski, P. W.; Radonic, A.; Kurth, A.; Nitsche, A., Genome-wide comparison of cowpox viruses reveals a new clade related to Variola virus. *PloS one* **2013,** 8, (12), e79953.
6. Chen, N.; Danila, M. I.; Feng, Z.; Buller, R. M.; Wang, C.; Han, X.; Lefkowitz, E. J.; Upton, C., The genomic sequence of ectromelia virus, the causative agent of mousepox. *Virology* **2003,** 317, (1), 165-86.
7. Mendez-Rios, J. D.; Martens, C. A.; Bruno, D. P.; Porcella, S. F.; Zheng, Z. M.; Moss, B., Genome sequence of erythromelalgia-related poxvirus identifies it as an ectromelia virus strain. *PloS one* **2012,** 7, (4), e34604.
8. Mavian, C.; Lopez-Bueno, A.; Bryant, N. A.; Seeger, K.; Quail, M. A.; Harris, D.; Barrell, B.; Alcami, A., The genome sequence of ectromelia virus Naval and Cornell isolates from outbreaks in North America. *Virology* **2014,** 462-463, 218-26.
9. Gubser, C.; Smith, G. L., The sequence of camelpox virus shows it is most closely related to variola virus, the cause of smallpox. *The Journal of general virology* **2002,** 83, (Pt 4), 855-72.
10. Li, G.; Chen, N.; Roper, R. L.; Feng, Z.; Hunter, A.; Danila, M.; Lefkowitz, E. J.; Buller, R. M.; Upton, C., Complete coding sequences of the rabbitpox virus genome. *The Journal of general virology* **2005,** 86, (Pt 11), 2969-77.
11. Tulman, E. R.; Delhon, G.; Afonso, C. L.; Lu, Z.; Zsak, L.; Sandybaev, N. T.; Kerembekova, U. Z.; Zaitsev, V. L.; Kutish, G. F.; Rock, D. L., Genome of horsepox virus. *Journal of virology* **2006,** 80, (18), 9244-58.
12. Likos, A. M.; Sammons, S. A.; Olson, V. A.; Frace, A. M.; Li, Y.; Olsen-Rasmussen, M.; Davidson, W.; Galloway, R.; Khristova, M. L.; Reynolds, M. G.; Zhao, H.; Carroll, D. S.; Curns, A.; Formenty, P.; Esposito, J. J.; Regnery, R. L.; Damon, I. K., A tale of two clades: monkeypox viruses. *The Journal of general virology* **2005,** 86, (Pt 10), 2661-72.
13. Garcel, A.; Crance, J. M.; Drillien, R.; Garin, D.; Favier, A. L., Genomic sequence of a clonal isolate of the vaccinia virus Lister strain employed for smallpox vaccination in France and its comparison to other orthopoxviruses. *The Journal of general virology* **2007,** 88, (Pt 7), 1906-16.
14. Meisinger-Henschel, C.; Schmidt, M.; Lukassen, S.; Linke, B.; Krause, L.; Konietzny, S.; Goesmann, A.; Howley, P.; Chaplin, P.; Suter, M.; Hausmann, J., Genomic sequence of

chorioallantois vaccinia virus Ankara, the ancestor of modified vaccinia virus Ankara. *The Journal of general virology* **2007,** 88, (Pt 12), 3249-59.

15. Osborne, J. D.; Da Silva, M.; Frace, A. M.; Sammons, S. A.; Olsen-Rasmussen, M.; Upton, C.; Buller, R. M.; Chen, N.; Feng, Z.; Roper, R. L.; Liu, J.; Pougatcheva, S.; Chen, W.; Wohlhueter, R. M.; Esposito, J. J., Genomic differences of Vaccinia virus clones from Dryvax smallpox vaccine: the Dryvax-like ACAM2000 and the mouse neurovirulent Clone-3. *Vaccine* **2007,** 25, (52), 8807-32.
16. Goebel, S. J.; Johnson, G. P.; Perkus, M. E.; Davis, S. W.; Winslow, J. P.; Paoletti, E., The complete DNA sequence of vaccinia virus. *Virology* **1990,** 179, (1), 247-66, 517-63.
17. Fleischauer, C.; Upton, C.; Victoria, J.; Jones, G. J.; Roper, R. L., Genome Sequence and Comparative Virulence of Raccoonpox Virus: The First North American Poxvirus Sequence. *The Journal of general virology* **2015**.
18. Smithson, C.; Tang, N.; Sammons, S.; Frace, M.; Batra, D.; Li, Y.; Emerson, G. L.; Carroll, D. S.; Upton, C., The genomes of three North American orthopoxviruses. *Virus genes* **2016**.
